# Mutations in ARL2BP, a protein required for ciliary microtubule structure, cause syndromic male infertility in humans and mice

**DOI:** 10.1371/journal.pgen.1008315

**Published:** 2019-08-19

**Authors:** Abigail R. Moye, Nicola Bedoni, Jessica G. Cunningham, Urikhan Sanzhaeva, Eric S. Tucker, Peter Mathers, Virginie G. Peter, Mathieu Quinodoz, Liliana P. Paris, Luísa Coutinho-Santos, Pedro Camacho, Madeleine G. Purcell, Abbie C. Winkelmann, James A. Foster, Elena N. Pugacheva, Carlo Rivolta, Visvanathan Ramamurthy

**Affiliations:** 1 Department of Ophthalmology, West Virginia University, Morgantown, United States of America; 2 Department of Biochemistry, West Virginia University, Morgantown, United States of America; 3 Department of Computational Biology, Unit of Medical Genetics, University of Lausanne, Lausanne, Switzerland; 4 Rockefeller Neurosciences Institute, West Virginia University, Morgantown, WV, United States of America; 5 Department of Ophthalmology, Instituto de Oftalmologia Dr Gama Pinto, Lisbon, Portugal; 6 Department of Biology, Randolph-Macon College, Ashland, VA, United States of America; 7 Department of Genetics and Genome Biology, University of Leicester, Leicester, United Kingdom; 8 Clinical Research Center, Institute of Molecular and Clinical Ophthalmology Basel (IOB), Basel, Switzerland; 9 Department of Ophthalmology, University Hospital Basel, Switzerland; Monash University, AUSTRALIA

## Abstract

Cilia are evolutionarily conserved hair-like structures with a wide spectrum of key biological roles, and their dysfunction has been linked to a growing class of genetic disorders, known collectively as ciliopathies. Many strides have been made towards deciphering the molecular causes for these diseases, which have in turn expanded the understanding of cilia and their functional roles. One recently-identified ciliary gene is *ARL2BP*, encoding the ADP-Ribosylation Factor Like 2 Binding Protein. In this study, we have identified multiple ciliopathy phenotypes associated with mutations in *ARL2BP* in human patients and in a mouse knockout model. Our research demonstrates that spermiogenesis is impaired, resulting in abnormally shaped heads, shortened and mis-assembled sperm tails, as well as in loss of axonemal doublets. Additional phenotypes in the mouse included enlarged ventricles of the brain and situs inversus. Mouse embryonic fibroblasts derived from knockout animals revealed delayed depolymerization of primary cilia. Our results suggest that ARL2BP is required for the structural maintenance of cilia as well as of the sperm flagellum, and that its deficiency leads to syndromic ciliopathy.

## Author summary

The flagellated tails of sperm cells require a stringent developmental process that is essential for motility and fertility. The components that comprise the sperm tail assemble in regulated steps with protein processing, transport, and structural assembly dependent on each other for sperm tail maturity. In this work, we have identified ARL2BP, a previously retinal-associated protein, to be essential for sperm tail development and assembly. We show that without functional ARL2BP in humans or mice, sperm tails fail to develop, starting with the assembly of the core microtubular structure within the tail. Loss of ARL2BP also effects other ciliated cells, indicating a unique role for ARL2BP in ciliary microtubule formation. This research on ARL2BP provides further understanding on the links between vision and fertility. This work also demonstrates how genomic studies for human patients and murine models can coincide to provide greater insight into disease.

## Introduction

Cilia are short, protruding organelles often referred to as “signaling hubs”. These microtubule-based structures are involved in diverse functional roles, and impairment of their structure or function often leads to a class of genetic diseases known as “ciliopathies” [1]. Cilia contain a highly organized structure, consisting of a 9+0 (motile and immotile) or a 9+2 (motile) microtubule arrangement, starting with triplet tubules at their base (basal body and transition zone), doublet tubules throughout the axoneme, and singlets at their tip [2, 3]. Despite the retention of this core structure throughout the body, cilia in each tissue are modified to impart unique functionality, a feature that reflects the broad range of ciliopathy phenotypes. For instance, the cilium of photoreceptors, the light-sensing neurons of the retina, has the function of both connecting the different segments of the cell (the inner and the outer segment) and of allowing the transport of proteins and metabolites across these compartments [4]. Meanwhile, cilia present in the embryonic node during gastrulation can bend and sense fluid-flow, the event responsible for the left/right-patterning of the visceral organs [5]. On the other hand, the sperm flagellum is the longest in the body (~100μm in mice) and possesses additional accessory structures not present in other cilia (illustrated in S2 Fig), including the transient microtubular-based structure, the manchette, which assists in axoneme growth [6].

Ciliary defects have been associated with retinitis pigmentosa (RP), a progressive form of retinal degeneration leading to loss of photoreceptor cells and vision. A recent study has associated nonsense mutations in the polyglutamylase gene *TTLL5* with retinal degeneration and male infertility [7]. In support of these findings, *Ttll5* knockout mice, in addition to visual impairment, show flagella that are detached from the sperm head, disrupted axoneme patterns with loss of tubulin doublets, and a severe loss of motility in sperm cells [8, 9]. Similarly, defects in the Intraflagellar Transport Protein 27 (IFT27) are linked with the RP-associated Bardet-Biedl syndrome and cause sperm malformations leading to infertility in mice [10, 11].

One protein previously linked to RP is the ADP-Ribosylation Factor Like 2 Binding Protein (ARL2BP) [12–14]. In agreement with the cilia-related phenotypes, ARL2BP localizes to the connecting cilia of photoreceptor cells [15], and our previous findings showed that loss of ARL2BP results in abnormal doublet microtubule structure of the axoneme and shortened cilia in photoreceptor cells [16].

In this study, we report the identification of two homozygous mutations in the gene *ARL2BP* in three Portuguese patients from two consanguineous families displaying RP and male infertility. The murine knockout model for the same gene showed similar phenotypes, including retinal degeneration, immotile sperm cells and impaired spermatogenesis, as well as situs inversus and increased brain ventricular volume. Our data highlight a novel ciliopathic entity linking two structurally similar, yet functionally different, ciliary organelles—the photoreceptor connecting cilium and the spermatozoon flagellum, associating vision and the reproductive system.

## Results

### Clinical evaluation

**Fig 1:**
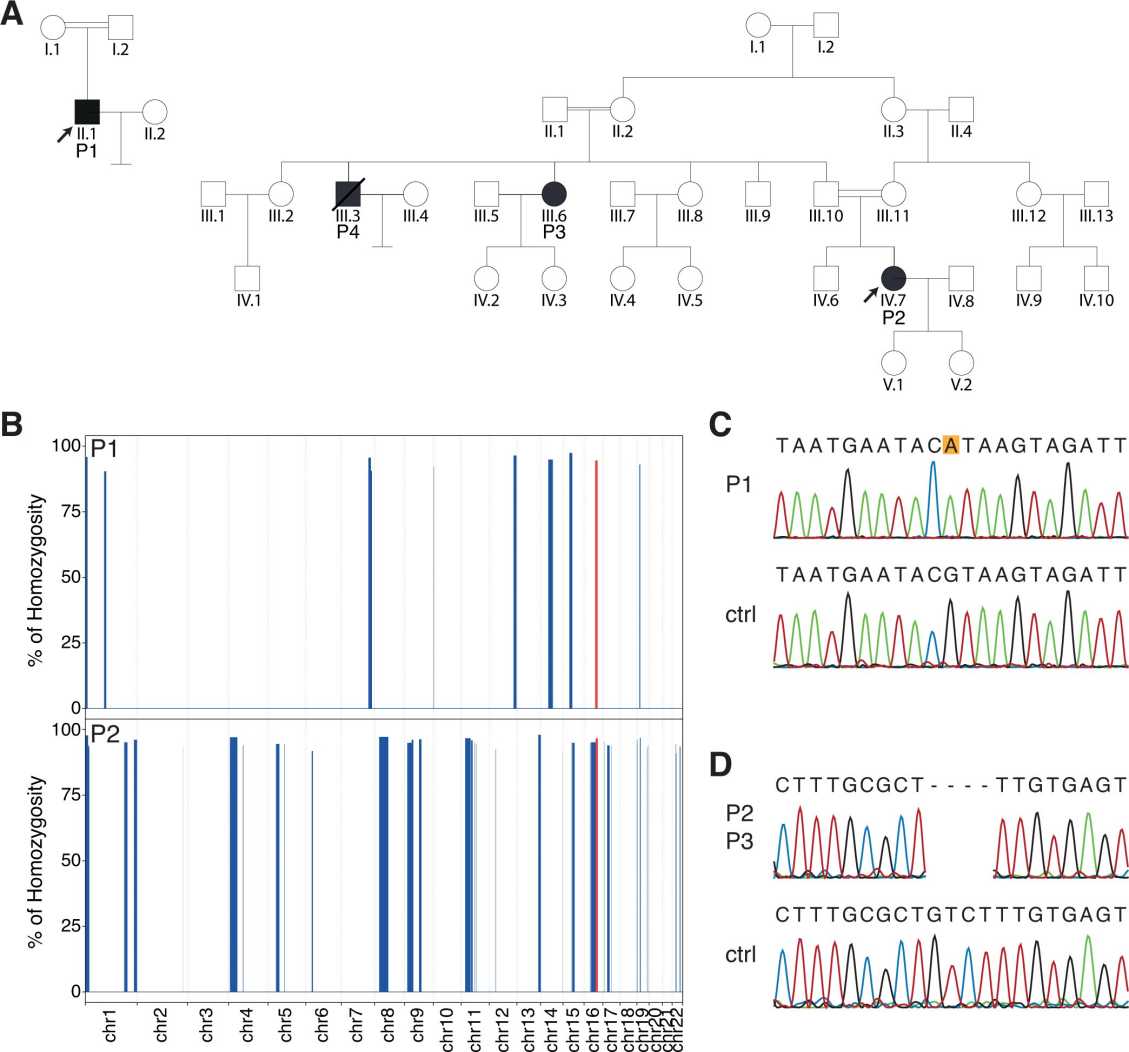
Pedigrees and genetic findings. (A) Pedigrees of the patients analyzed in this study. DNA was available only for subjects P1, P2, and P3. (B) Exome-wide homozygosity mapping for autosomal chromosomes, using the AutoMap tool. The autozygous region containing the gene *ARL2BP* is highlighted in red. (C and D) Sanger validation of the WES findings, showing the presence of a homozygous splice site mutation in patient P1 (**C**, NM_012106.3:c.207+1G>A, leading to p.Asp35PhefsTer8), and a frameshift deletion in patients P2 and P3 (**D**, NM_012106.3:c.33_36delGTCT: p.Phe13ProfsTer15), alongside with relevant control sequences (ctrl).

The index subject P1 (ID: LL1) was initially evaluated at age 40 and diagnosed with retinitis pigmentosa. Born from a consanguineous union (Fig 1A), and originating from Portugal, he was first diagnosed with myopia at age 8 and developed night blindness and photopsia by the age of 26. Over the following 10 years, he developed a progressive loss of vision and bilateral constriction of the peripheral visual fields. The patient noticed a more pronounced reduction in visual acuity at age 37, associated with onset of photophobia (both eyes), and later, metamorphopsia in the right eye.

**Fig 2:**
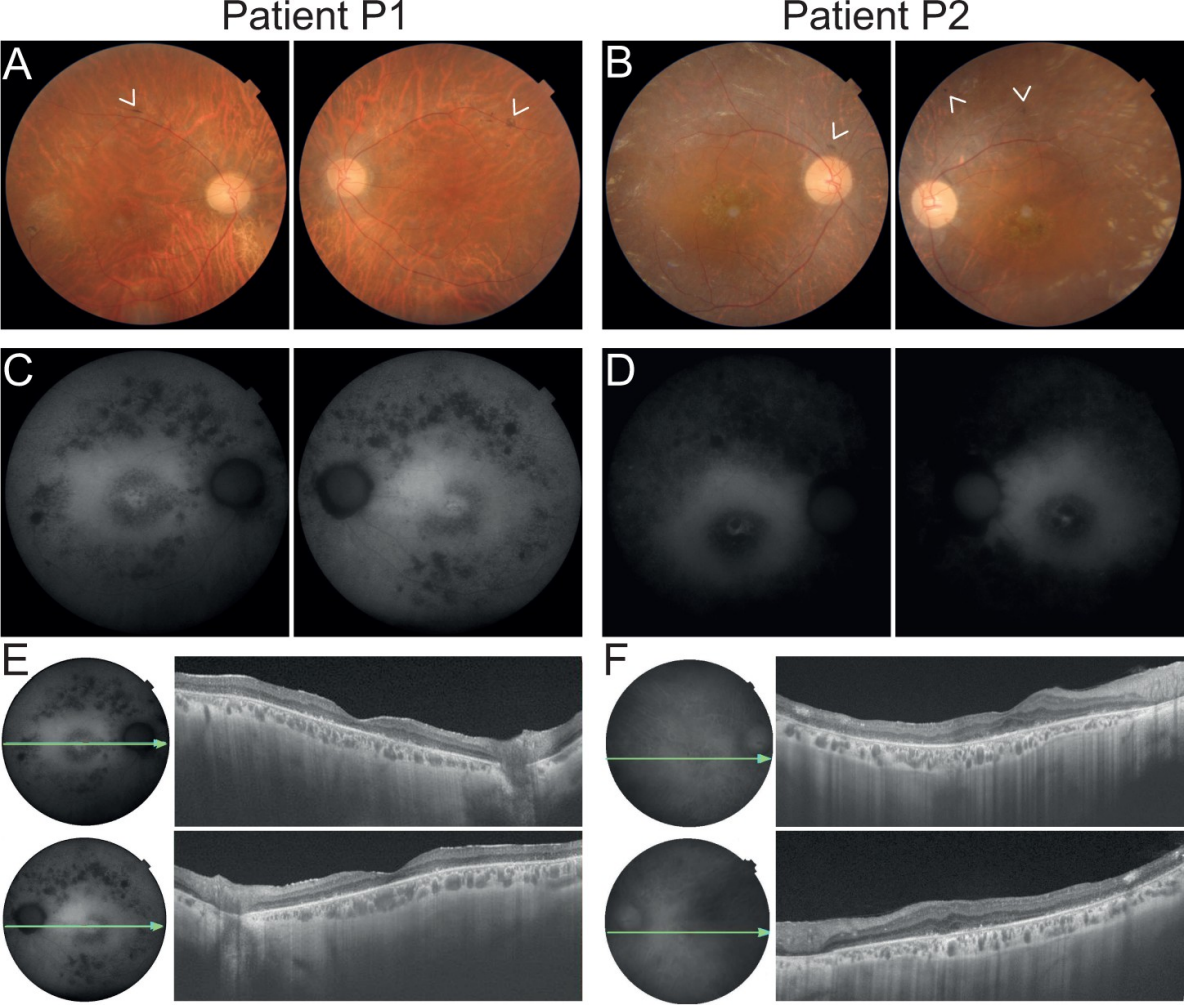
Findings on retinal imaging of patients P1 and P2. (A and B) Fundus photos of patients P1 and P2, respectively. P1 presents a pale optic disc, vascular thinning and retinal atrophy in the posterior pole and along the vascular arcades. Scarce pigment deposition can be found along the superior vascular arcade (as shown by the arrowheads). Similarly, P2 displays a pale optic disc, vascular thinning and marked retinal changes, with opalescent areas of the retinal tissue in the periphery and central atrophic lesion. Scarse pigment deposition can also be found adjacent to the optic nerve head and periphery (arrowheads). (C and D) Fundus autofluorescence showing in P1 multiple hypoautofluorescent spots in the periphery corresponding to RPE atrophy, which in contrast is largely diffused in P2. Moreover, both patients present a central hyperautofluorescent ring, typical of RP. (E and F) SD-OCT revealing in both patients diffuse retinal thinning, absent photoreceptors and enhanced visualization of the choroidal vessels. Panels A-D: right eye followed by left eye. Panels E and F: right eye above and left eye below.

At age 42, his best corrected visual acuity (BCVA) was 0.2 in the right eye (OD) and 0.25 in the left eye (OS). Slit-lamp examination revealed moderate opacities of the crystalline lenses and generalized chorioretinal atrophy, including punched-out lesions, affecting the periphery and the posterior pole, upon dilated fundus examination (Fig 2A). Scarce pigment deposits were visible in the peripheral retina as well as bilateral optic disc pallor and attenuated retinal vessels (defining the characteristic triad of retinitis pigmentosa). Fluorescence angiography showed generalized window defects and atrophy of the photoreceptor/pigment epithelium complex. Fundus autofluorescence showed spotty areas of hypo-autofluorescence outside of the vascular arcades and a central ring of hyper-autofluorescence in the posterior pole (Fig 2C). At last examination, at age 43, marked progression of peripheral visual field constriction was noted in both eyes, which was more severe in the OD (limited to the central 10–15 degrees). Spectral domain optical coherence tomography (SD-OCT) revealed pronounced atrophy of the retinal layers, with enhanced visualization of the choroidal vessels (Fig 2E). Electroretinograms (ERG) showed no response to light stimuli, either from cone or from rod photoreceptor cells (S1 Fig).

**Fig 3:**
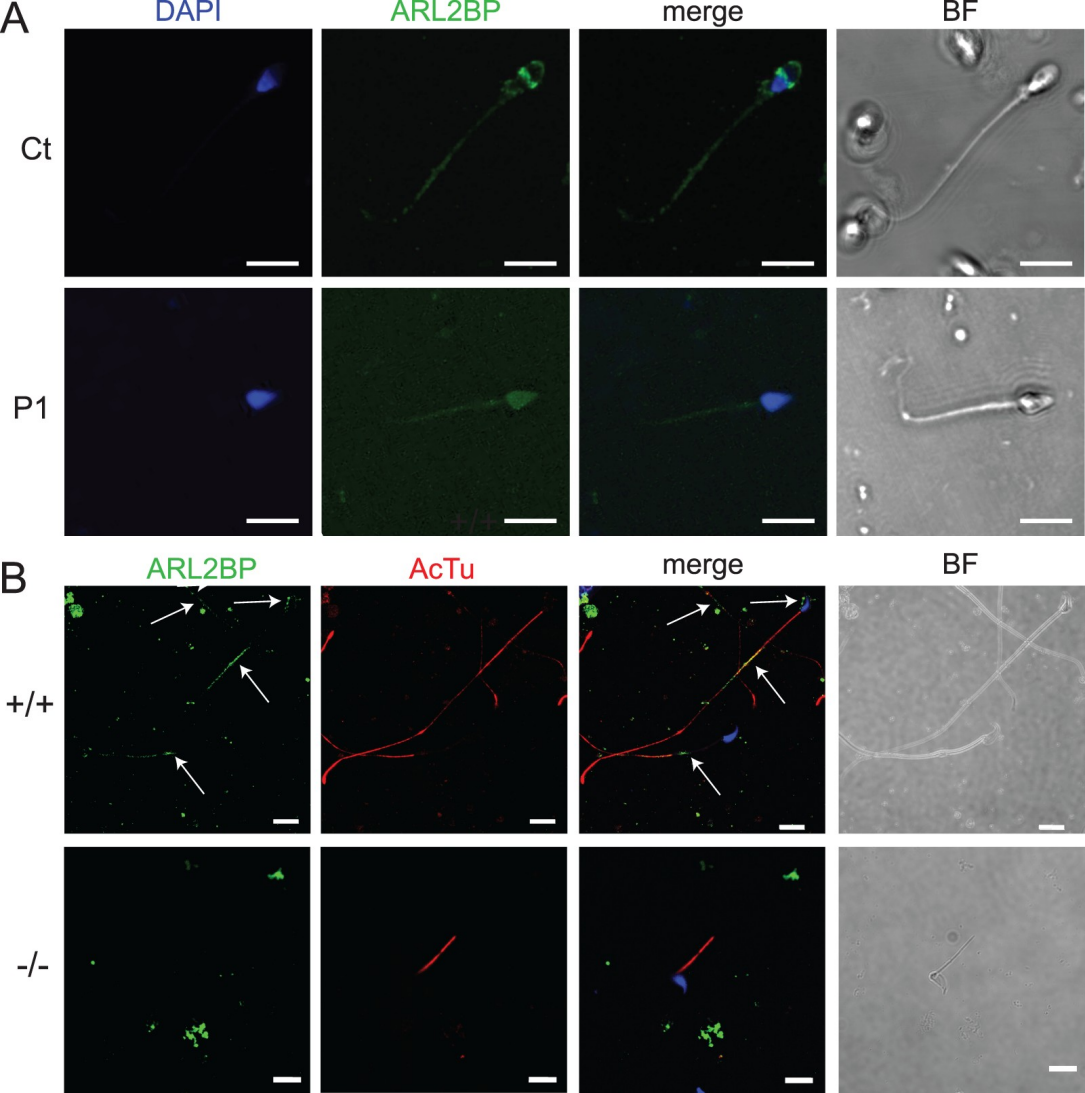
ARL2BP localizes to the sperm tail in humans and mice. (A) Staining for ARL2BP (green) and DNA (blue) in a sperm sample from a control individual (top) and from patient P1 (bottom). (B) WT (+/+) and KO (-/-) murine sperm stained for ARL2BP (green) and Acetylated Tubulin (AcTu, red). Lack of ARL2BP staining in the KO serves as a negative control. Scale Bar = 10μm.

The patient’s past medical history revealed cardiac arrhythmia under bisoprolol treatment and infertility. Due to unsuccessful conception, a full spermiogram analysis was performed, the diagnosis resulting in severe asthenozoospermia (complete absence of motility). Table 1 summarizes this analysis, as compared to the reference normal values described by the World Health Organization [17]. The causes of infertility were investigated by exploring possible occurrence of deletions in the Y chromosome, as described for the Sertoli cell-only syndrome (OMIM: 305700). The presence of 21 genetic markers (STS) on chromosome Yq and two markers on Yp was verified (S1 Table), therefore excluding this possibility. A fresh sperm sample, originally collected for immunofluorescence analysis for the present study, showed that most sperm heads were detached from flagellum, and approximately 80% of intact cells had a shorter flagellum (Fig 3A). Nevertheless, the percentage of normal sperm formed was within standard WHO reference values.

**Table 1:** Spermiogram of patient P1.

	P1	Lower reference limit^A^
Semen volume (ml)	1	1.5
Sperm concentration (10^6^/ml)	50	15
Total number (10^6^/ejaculate)	50	39
Total motility^B^ (%)	0	40
Normal forms (%)	8	4
pH	9	7.2

^A^ Reference data from WHO [17]

^B^ Total motility includes progressive and non-progressive motility

Patient P2 (ID: LL89), a woman, was diagnosed with a classical form of retinitis pigmentosa at the age of 36 years. The patient was born from a consanguineous union between first degree cousins of Portuguese origin (Fig 1A). Initial visual complaints started with photophobia at 11 years of age and progressed to night blindness and photopsia by the age of 25. Constriction of the peripheral visual field was noted at the age of 28. Disease progression led to a sharp reduction in visual acuity at age 38 and to loss of color vision by the age of 40. The patient underwent cataract surgery at age 40 and 48. At 50 she developed metamorphopsia in the OS. At the last ophthalmologic examination, patient P2 showed a BCVA of 0.3 in the OD, 0.16 in the OS, and severe constriction of the peripheral visual field, with bilateral tunnel-like vision, restricted to the 5 degrees central. Dilated fundus examination revealed attenuated retinal vessels, pale optic discs (Fig 2B) and a marked generalized chorioretinal atrophy in the posterior pole and in the periphery, with areas of complete atrophy in the inferior peripheral region of the right eye. Mild to moderate mottled pigment deposition in the retinal periphery, along with some irregular white patches in the posterior pole near the superior vessels, was also observed. Fundus autofluorescence was notable for generalized hypo-autofluorescence with a wide hyper-autofluorescent ring in the posterior pole (Fig 2D). SD-OCT revealed pronounced atrophy of the outer retinal layers and the presence of a foveal cyst (Fig 2F). Patient P2’s paternal aunt (P3) and her uncle (P4) were also diagnosed with RP, showing similar progression and manifestations. Specifically, P3 began to be visually impaired in her 20s with the onset of night blindness. Her visual field progressively shrank for both eyes and considerably worsened after the birth of her two daughters in her 30s, evolving to a state of complete blindness by the age of 50.

There is no clinical history of other ciliopathic disorders in this family, with the exception of P4, who suffered from chronic bronchitis. This latter patient died of multiple myeloma prior to this study, thus preventing further clinical analysis. Interestingly, P4 never conceived any offspring in spite of multiple attempts (as reported by his sister, patient P3), whereas both female patients P2 and P3 had two healthy children.

None of the patients in this study had self-reported respiratory conditions or subjective complaints (except for P4), metabolic disturbances, situs inversus or skeletal abnormalities.

### Identification of pathologic variants in the *ARL2BP* gene

The DNA of P1 was initially screened for mutations in the *TTLL5* gene, due to the similarities in phenotype, but the results were negative. Following whole-exome sequencing (WES), the data were analyzed using an internal *in silico* pipeline assessing variant frequency in the general population, quality, and predicted impact at the protein level. This filtering resulted in 7 homozygous changes, of which 6 resided in autozygous regions (Fig 1B). Among these 6 variants, a single-nucleotide substitution disrupting the canonical consensus donor splice site downstream of exon 3 in *ARL2BP* (NM_012106.3:c.207+1G>A, hg19) was identified (Fig 1C).

P2 was studied using the same methodology, and 20 of the 23 rare homozygous variants resulting from our pipeline were located in autozygous regions. These included a 4-bp deletion in the coding sequence of *ARL2BP* exon 1, resulting in a frameshift and creation of an early stop codon at the beginning of exon 2 (c.33_36delGTCT:p.Phe13ProfsTer15) (Fig 1C and 1D). P3 was also confirmed to carry the same homozygous mutation (Fig 1D).

### *ARL2BP* aberrant splicing in patient P1

**Fig 4:**
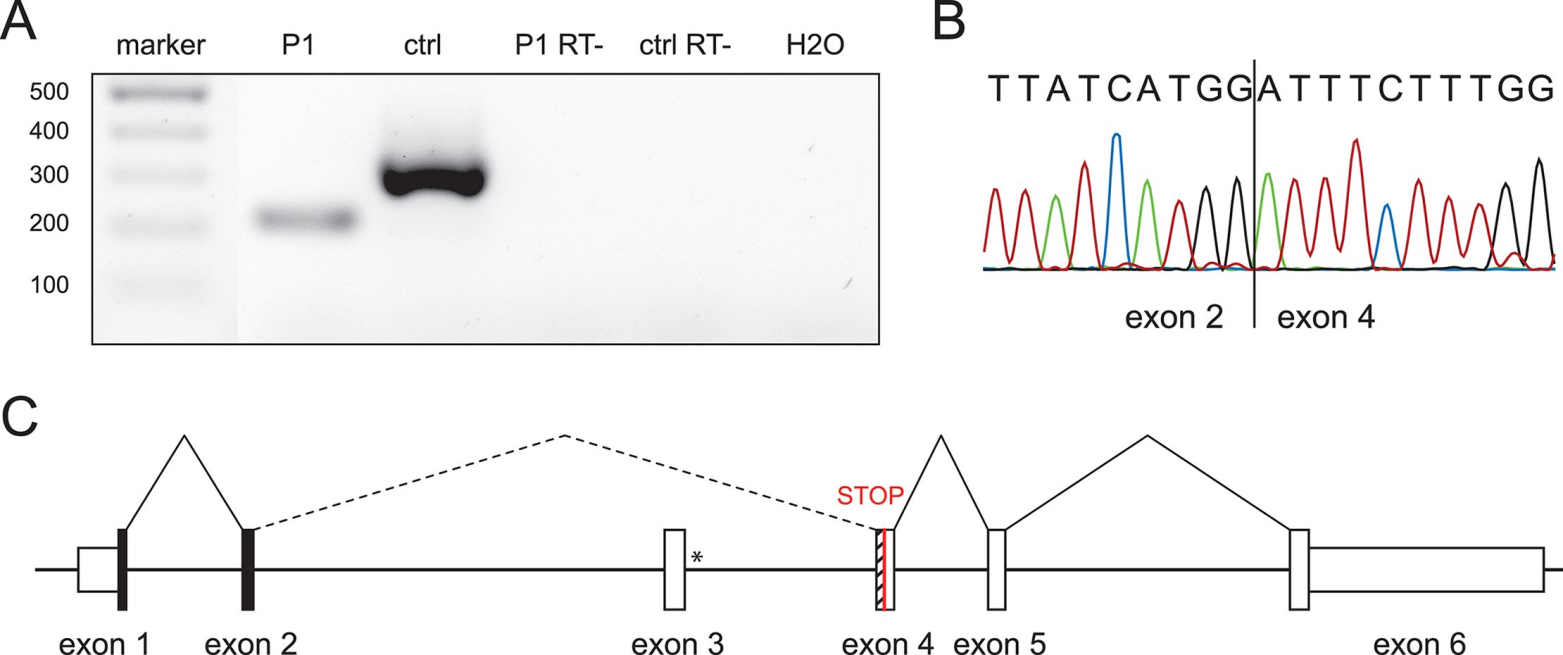
Effect of the splice site mutation in patient P1. (A) Agarose gel showing the RT-PCR amplified product of *ARL2BP* spanning exon 2 to 4 using cDNA obtained from sperm-derived RNA (P1, cDNA from patient; ctrl, cDNA from a healthy control; P1 RT- and ctrl RT-, reverse transcription reaction lacking the RT enzyme with templates from P1 and a ctrl, respectively; H2O, control reaction with no cDNA template) (B) Chromatogram of the PCR product from patient P1, from panel (A). (C) In-scale schematic representation of the splicing pattern resulting from the presence of the mutation c.207+1G>A that leads to the skipping of exon 3 and the joining of exon 2 with exon 4, with subsequent shifting of the reading frame and creation of a premature stop codon in exon 4 (red). The asterisk denotes the location of the mutation. The in-frame exons are in black, whereas the striped portion of exon 4 indicates the out-of-frame region.

RT-PCR analysis was performed on cDNA derived from P1’s sperm RNA and compared with RNA from a healthy control donor. Gel electrophoresis revealed the absence of a band corresponding to the expected PCR product in P1, and the appearance of a smaller fragment (Fig 4A). Sanger sequencing of this latter fragment showed that in P1’s sperm *ARL2BP* transcripts were abnormally spliced, and that c.207+1G>A caused the skipping of exon 3, the fusion of exon 2 with exon 4, and the shift of the canonical reading frame, leading to premature termination at the 9^th^ codon of exon 4 (Fig 4B and 4C).

### ARL2BP localization in human sperm

To determine the role for ARL2BP in spermiogenesis, the final stages of spermatogenesis, we investigated the localization of ARL2BP within the sperm cell. In the human control sample, ARL2BP localized at the base of the flagellum, as well as at the equatorial zone of the sperm head (Fig 3A). In cells from P1, we observed most of the sperm heads separated from their tails, though some intact sperms had a shortened tail. In the majority of these cells, staining for ARL2BP was non-specific with faint background (Fig 3A). In approximately 2–5% of the spermatozoa from P1, staining was similar to that of the control sample, suggesting residual expression of the wildtype, correctly spliced ARL2BP isoform in this patient, which often occurs for mutations affecting splicing sites [18–21].

In agreement with findings from human samples, ARL2BP was found in the sperm head at the head-tail connecting apparatus (HTCA) and principal piece in murine sperm (Fig 3B). In contrast, no signal was observed in KO murine sperm, demonstrating the specificity of the antibody used.

### Immotile sperm with stubby tails and a decreased sperm cell count in P1 and in *Arl2bp* KO mice

**Fig 5:**
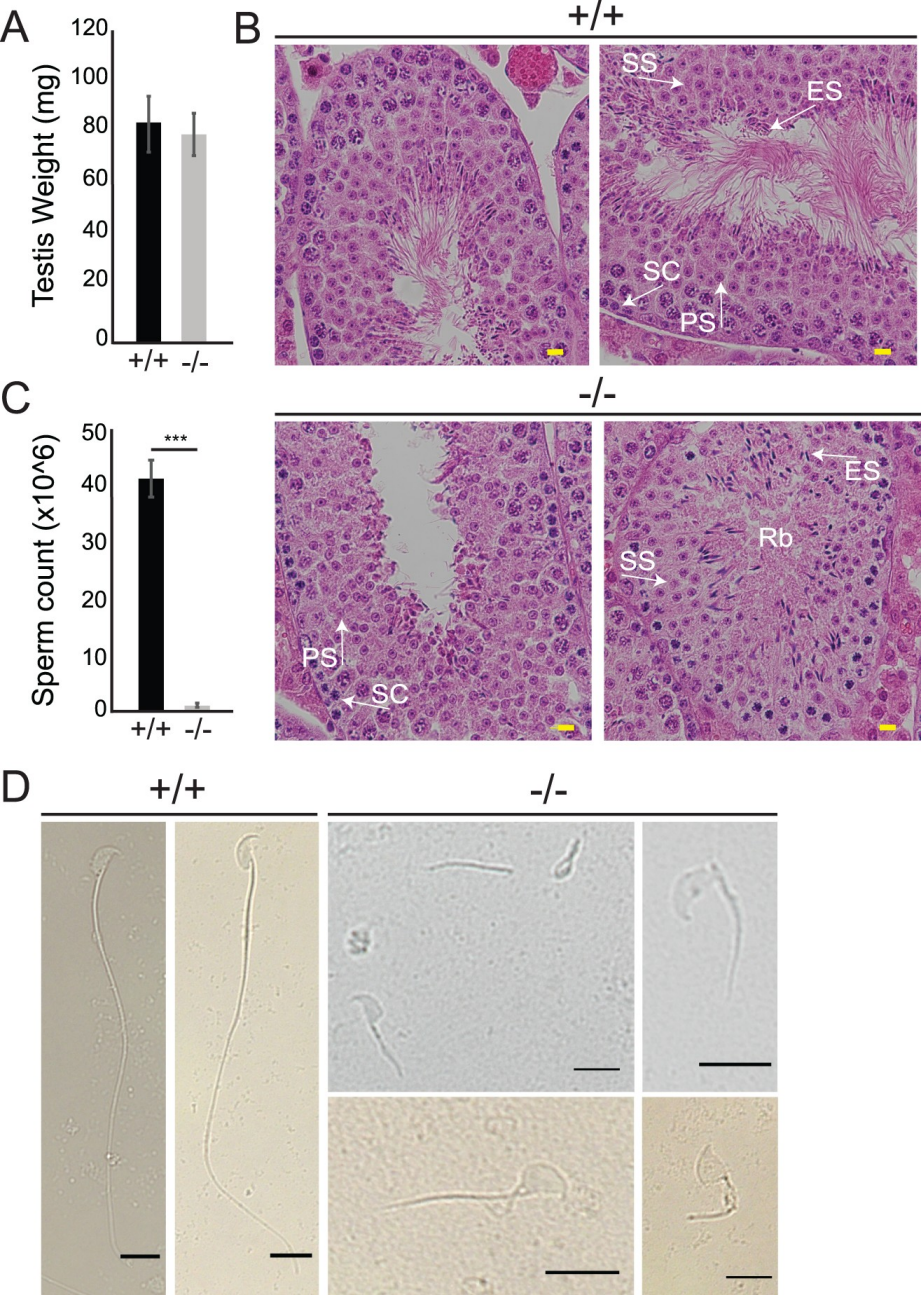
Loss of ARL2BP leads to decreased sperm count and abnormal sperm structure in mice. (A) The average weight of testis between WT (+/+) and KO (-/-) mice are comparable, according to unpaired, two-tailed *t* test (*n* = 10). Data are represented as the mean ± SEM. (B) H&E sections of WT (+/+) and KO (-/-) testis. Residual bodies (Rb), PS = primary spermatocyte, SS = secondary spermatocyte, ES = elongating spermatid, SC = sertoli cells. Scale Bar = 20μm. (C) Graph presenting the epididymal sperm cell counts of WT (+/+) and KO (-/-) mice. Data are represented as the mean ± SEM. ***P = 0.0002, according to unpaired, two-tailed *t* test (*n* = 3). (D) Light microscopy images displaying WT (+/+) sperm with normal sperm structure, while KO (-/-) sperm show abnormal sperm heads, shorter sperm tails, detached head and tails, and retained cytoplasm. Scale Bar = 10μm.

Concurrent with the identification of the male patient, we discovered that male *Arl2bp* KO mice were infertile, as they were not yielding any litters during the generation of the murine *Arl2bp* KO model to study blindness [16]. Therefore, we examined sperm motility with live imaging and found that the KO sperm were immotile, in agreement with the human patient phenotype (S1 and S2 Videos). Of note, testis size and weight were comparable between WT and KO (Fig 5A), and Heterozygous males were comparable to WT in every murine examination performed throughout this study. Morphological analysis of the testes by H & E staining revealed normal spermatogenesis in KO animals. However, sperm release into the lumen appeared impaired, with a smaller lumen area, an absence of sperm tails, and an increase in residual bodies (RB) (Fig 5B). Inspection of the murine KO sperm revealed a drastically decreased epididymal sperm cell count (Fig 5C), and additional light microscopy images of cauda epididymis sperm revealed that all *Arl2bp* KO sperm had gross morphological defects, including numerous detached heads and tails, kinked necks, bent tails, stubby tails, abnormal heads, and cytoplasmic bulges attached to the tails (Fig 5D). These results are consistent with a high incidence of sperms with abnormal morphology (92%) reported in patient P1 (Table 1, Fig 4A).

### Failure to complete spermiogenesis in the absence of ARL2BP

**Fig 6:**
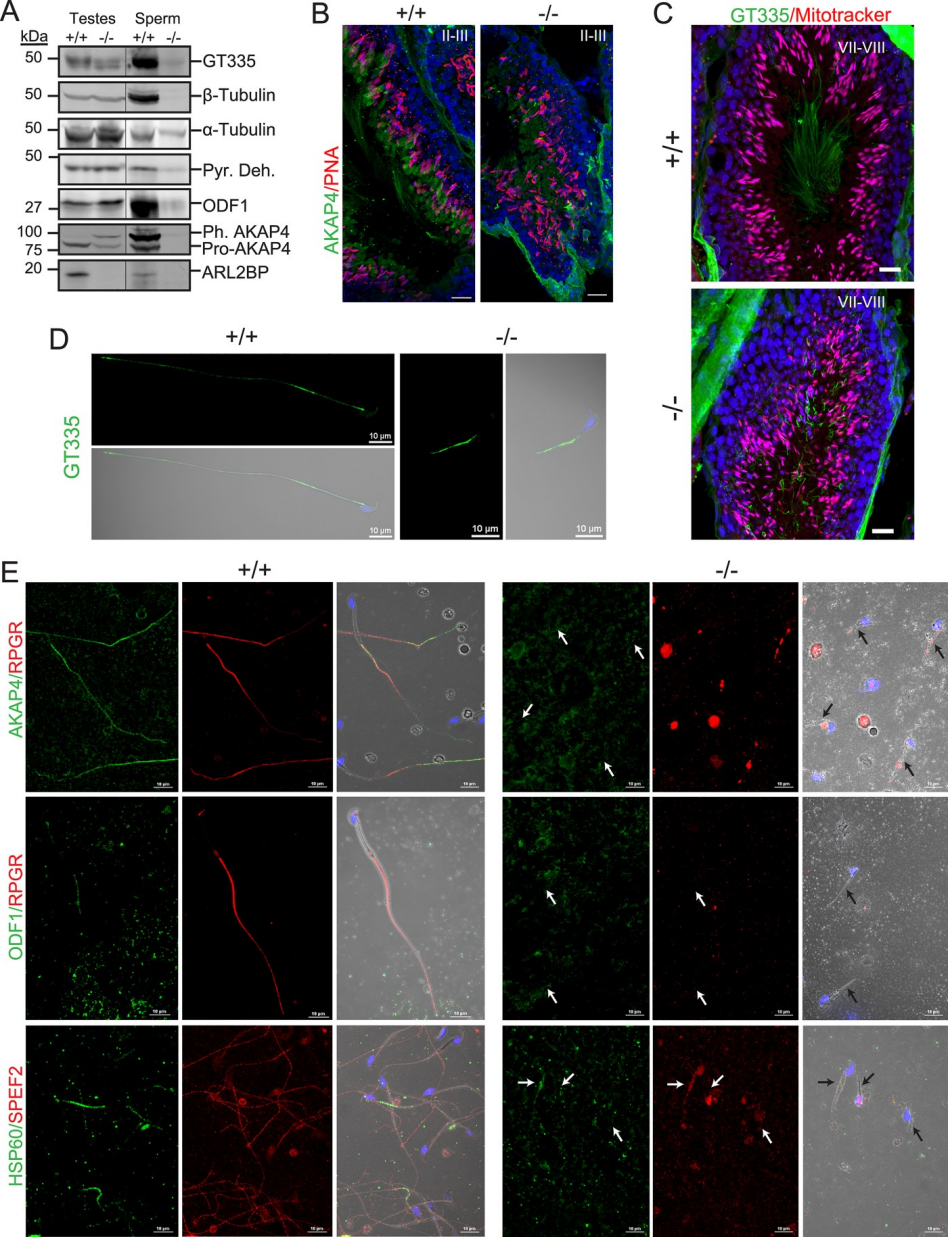
ARL2BP loss results in impaired sperm tail development. (A) Immunoblot of WT (+/+) and KO (-/-) testis and sperm lysates probed for the indicated sperm tail markers: axonemal markers (Glutamylated tubulin (GT335) and β-tubulin) and accessory structure markers (Pyruvate dehydrogenase (Pyr. Deh.), Outer Dense Fiber 1 (ODF1), and A-kinase anchoring protein 4 (pre-processed = Pro-AKAP4 and phosphorylated, processed = Ph. AKAP4). Molecular weights in kilodaltons (kDa) are displayed on the left. α-tubulin is used as the loading control. (B) WT (+/+) and KO (-/-) murine testes sections stained with PNA lectin (acrosomes, red) and AKAP4 (green), or (C) mitotracker (mitochondria, red) and GT335 (green). The nuclei are stained with DAPI (blue). Scale Bar = 20μm. (D and E) Sperm stained with the indicated sperm tail markers in WT (+/+) and KO (-/-) murine sperm. GT335 –axoneme, green; AKAP4 –fibrous sheath, green; Retinititis Pigmentosa GTPase Regulator (RPGR–axoneme, red); ODF1 –outer dense fibers, green; Heat Shock Protein 60kDa (HSP60 –mitochondrial sheath, green), and Sperm Flagellar Protein 2 (SPEF2 –axoneme, red). Arrows point to sperm tails. Scale Bar = 10μm.

The severity of sperm immotility in the KO mice spurred the investigation into the morphology and formation of the sperm tail and accessory structures. We first examined the sperm tail core (axoneme) using microtubule-associated markers (antibodies listed in Table 2). Despite severe loss in protein levels in sperm lysates from *Arl2bp* KO animals (Fig 6A), Acetylated Tubulin (AcTu) and Glutamylated Tubulin (GluTu, GT335) were found in the sperm tail in WT and KO animals (Figs 3B and 6D). Interestingly, testes cross-sections from KO mice stained with GluTu revealed a further irregularity in tail shapes, as the tails were spiraled (Fig 6C). Retinitis Pigmentosa GTPase Regulator (RPGR) and Sperm Flagellar Protein 2 (SPEF2), both axoneme-associated proteins, displayed highly diminished and spotty staining in KO murine sperm (Fig 6E). These findings show that loss of ARL2BP does not interrupt the initiation of microtubular axoneme growth. However, shortened axonemes and a decrease in axoneme-associated protein localization to the sperm tail indicates that maturation of the axoneme is impaired.

**Table 2:** Antibodies. Information relative to antibody origin and working dilutions are presented below.

Antibody	Catalog #	Area stained	Dilution IF	Dilution Immunoblot
Mouse anti-Acetylated Tubulin	Sigma Aldrich T6793	Connecting Cilium	1:1000	1:2000
Rabbit anti- RPGR	Gift from Dr. Hemant Khanna, University of Massachusetts	Connecting cilium	1:500	1:1000
Rabbit anti- Arl13b	Protein Tech Group 17711-1- AP	Primary Cilium	1:1000	1:1000
Mouse anti- Arl13b	Antibody Inc.	Primary Cilium	1:500	N/A
Mouse anti-γtubulin	Sigma Aldrich GTU-88	Basal body	1:200	N/A
Rabbit anti-Pericentrin	Abcam 4448	Basal body	1:200	N/A
Mouse anti-glutamylation	Adipogen GT335	Connecting Cilium	1:1000	1:2000
Rabbit anti-ARL2BP	In-house, [16]	IS, BB, CC	1:500	1:1000
Mouse anti-GAPDH	Fitzgerald 10R-G109a	N/A	N/A	1:10,000
Mouse anti-alpha tubulin	Sigma Aldrich T9026	N/A	N/A	1:2000
Mouse anti-beta tubulin	Sigma Aldrich T8328	N/A	N/A	1:2000
Mouse anti-AKAP4	sc-135827	Fibrous Sheath	1:250	1:500
Mouse anti-ODF1	sc-390152	ODF	1:250	1:500
Rabbit anti-SPEF2	HPA040343	CP	1:250	N/A
Mouse anti-HSP60	Enzo Life Sciences SPA-806	MS	1:250	N/A
Rabbit anti- Pyruvate Dehydrogenase	Thermo Fisher PA5-21536	Mitochondria	N/A	1:1000
Mitotracker	Life Technologies M7510	Mitochondria	100nM in media	N/A
Biotinylated Peanut Agglutinin (PNA)	Vector Laboratories	Acrosome	1:2000	N/A
4′,6-diamindino- 2- phenylindole (DAPI)	Invitrogen	Nuclei	1:2000	N/A

To determine if the assembly of the accessory structures is normal in the absence of ARL2BP, we assessed sperm tails using markers such as A-Kinase Anchoring Protein 4 (AKAP4, fibrous sheath, FS) and Outer Dense Fiber Protein 1 (ODF1, outer dense fiber). Both markers were absent in murine KO sperm, though present in murine KO testes lysates (Fig 6A and 6E). Additionally, both forms of AKAP4 were at the expected distribution in WT animals [22], with most of the soluble, non-assembled precursor to AKAP4 (pro-AKAP4, 82kDa) in the testes samples, while in the sperm lysates the phosphorylated form of AKAP4 assembled into the FS (Ph-AKAP4,109kDa) was the most represented (Fig 6A). In contrast, both forms of the protein were present in the KO testes, with neither present in the sperm lysates (Fig 6A). Supporting this finding, staining in KO testes sections revealed retention of AKAP4 in the residual bodies shed during spermiogenesis, and an absence of AKAP4 from sperm tails. In comparison, WT testes displayed AKAP4 in both residual bodies and luminal sperm tails (Fig 6B). Conversely, we observed staining for Heat-Shock Protein 60kDa (HSP60, mitochondrial sheath) in murine KO sperm (although in reduced amounts) (Fig 6E). This finding was independently confirmed by the presence of pyruvate dehydrogenase protein (Pyr Deh., mitochondrial sheath) in sperm lysates (Fig 6A). Furthermore, staining of WT and KO testes cross-sections using Mitotracker showed mitochondria in elongating spermatids (Fig 6C), indicating the formation of a mitochondrial sheath. All together, these results demonstrate a failure to complete spermiogenesis by the inability to form the outer dense fiber layer or assemble the fibrous sheath in *Arl2bp* KO animals.

### Structural abnormalities in acrosome with lack of ARL2BP

**Fig 7:**
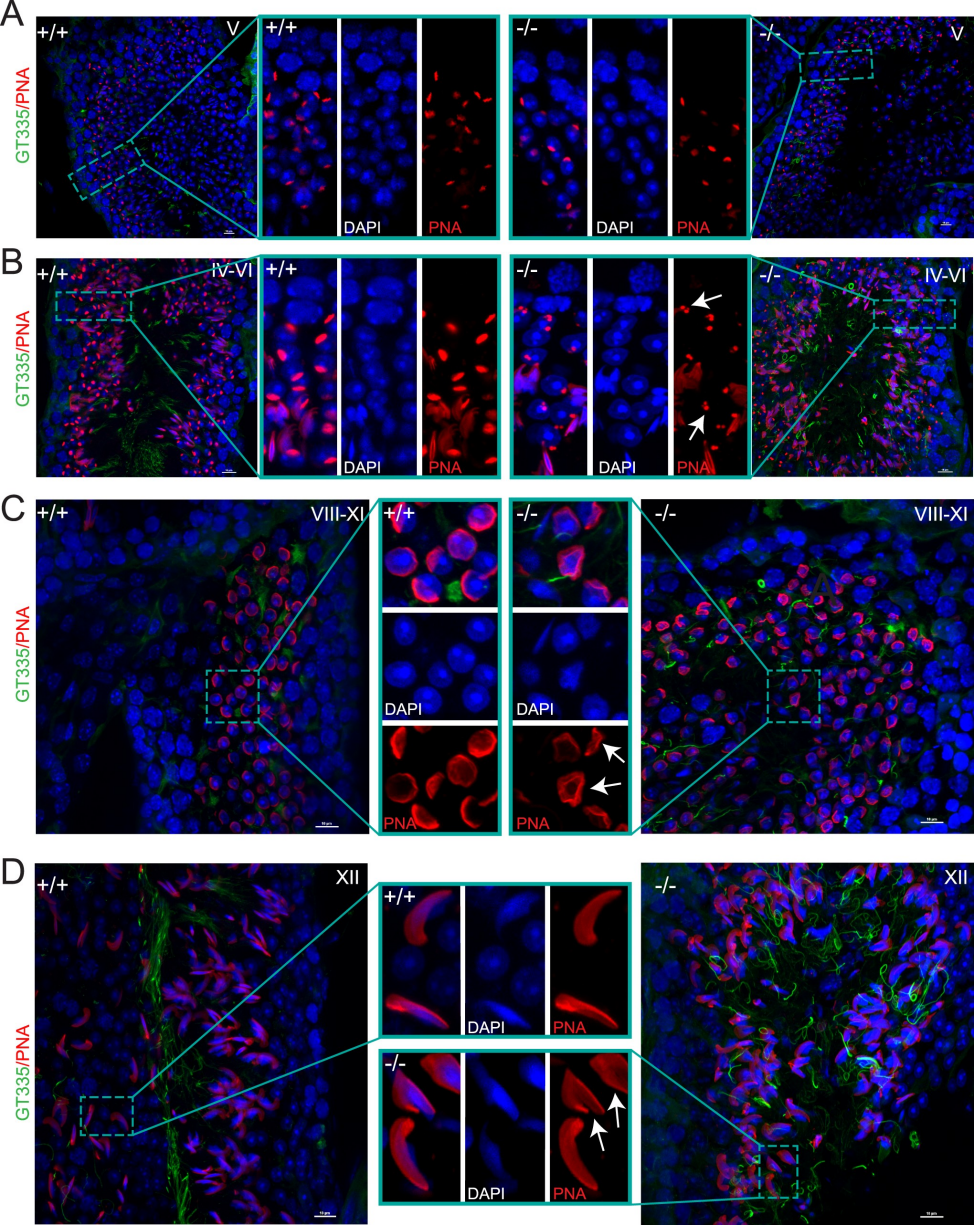
Abnormal acrosomes with loss of ARL2BP. (A-D) WT (+/+) and KO (-/-) adult murine testes sections displaying staining of DAPI (nuclei, blue), PNA lectin (acrosomes, red) and GT335 (green, sperm tails in stages IV-VI and XII). Scale bar = 10μm. Inserts show magnified image. Arrows point to abnormal acrosomes.

ARL2BP staining in the sperm head and the presence of abnormally shaped sperm heads in the male patient and KO mice prompted our investigation of acrosome development throughout spermatogenesis. The acrosome spreads like a cap around the nucleus in stages V-VII (cap phase), as observed in the WT testes (Fig 7A). However, KO animals displayed acrosomal irregularities exemplified by clusters of acrosomal granules instead of the cap structure (Fig 7B). Furthermore, at later stages in spermatogenesis, there were instances of irregularly shaped acrosomes surrounding nuclei. Fig 7C shows acrosome caps in KO testes at stages VIII-XI that are irregularly shaped instead of round, with a delicate appearance. While at stage XII, there were few misshapen acrosomes displaying a “molar tooth” shape (Fig 7D). Of note, most acrosomes on elongating spermatids in KO testes appear normal, suggesting that ARL2BP is not crucial for acrosome formation.

### ARL2BP is essential for flagellar microtubule structure

**Fig 8:**
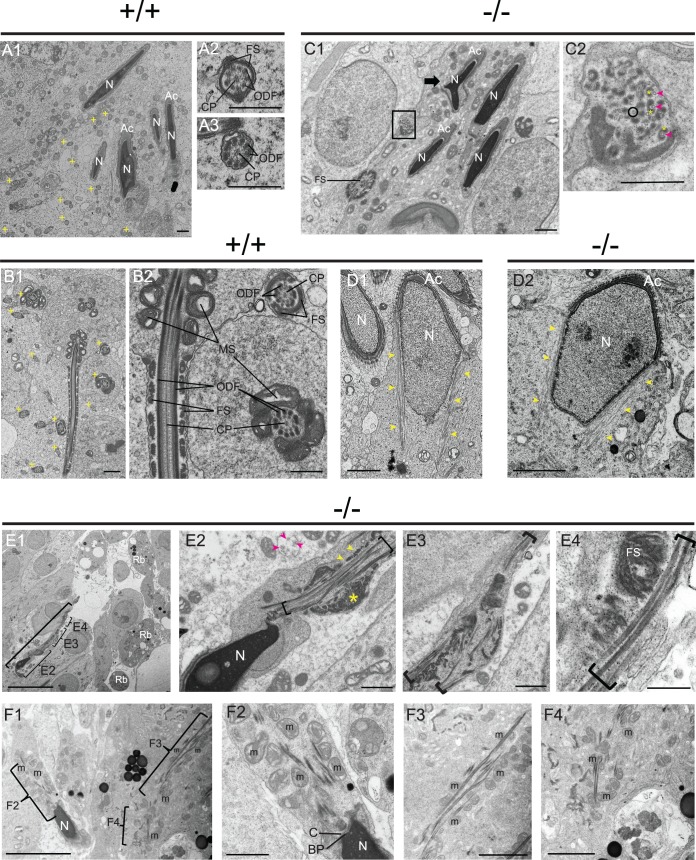
Loss of ARL2BP results in disorganized axoneme and accessory structures. (A1-A3) WT testes. Panel A1 showing multiple heads with normal nuclei (N) and acrosomes (Ac). Tail cross-sections (+). Scale Bar = 1 μm. (A2 and A3) tail cross-sections with central pair (CP) microtubules, outer dense fibers (ODF), and fibrous sheath (FS). Scale Bar = 500 nm. (B1) Tail cross-sections from WT testes (>20, +) with normal FS, ODF, CP, and mitochondrial sheath (MS). Scale Bar = 1 μm. (B2) Higher magnification of (B1) Scale Bar = 500 nm. (C1) KO spermatid heads mostly normal with some aberrantly shaped (arrow). Tail cross-sections show disorganized FS components. Scale Bar = 1 μm. (C2) Higher magnification of box in (C1) shows disorganized microtubules (arrowhead) with associated ODF material (*). Some microtubules are incomplete (circle). Scale Bar = 500 nm. (D) Step 9 spermatids with normal manchettes (arrowhead) in WT (D1) and KO (D2). Scale Bars = 1 μm. (E1) Abnormal spermatid in KO (bracket) with microtubules, ODF, and FS present but disorganized (Higher mag in E2, E3 Bar = 1 μm, and E4 Bar = 500 nm). Residual bodies (Rb) in the lumen. Bar = 10 μm. (E2) Abnormal head and neck region with parallel microtubules and disorganized tail accessory structures. Microtubules (bracket and pink arrowheads), putative ODF (yellow arrowheads), and putative FS (*). Tail cross-section top/center with single, unorganized microtubules (<). Bar = 1 μm. (F1-F4) KO testes show disorganized microtubules and aggregation of mitochondria (M) that fail to organize into a tight spiral. Bar = 5 microns; (F3 and F4) Bar = 2 microns. (F2) M and microtubules are disorganized. In the neck region, basal plate (BP) and capitulum (C) are seen. Bar = 1 μm.

Extensive analysis of ultrastructural images from WT and KO murine testes and epididymis tissue corroborated our earlier observations of decreased sperm count and lack of sperm tails in the KO. WT sperm tails demonstrated the organized structure illustrated in S2 Fig (Fig 8A and 8B). Additionally, spermatogenesis appeared to be relatively normal through early tail formation in spermiogenesis, including formation of the manchette with centrally located centrioles in both WT and *Arl2bp* KO (Fig 8C and 8D). However, significant abnormalities in head, neck, and tail ultrastructure were noted in later stages of spermiogenesis in *Arl2bp* KO animals. Longitudinal and cross-sections of *Arl2bp* KO sperm tails revealed that microtubules were present in parallel arrays in the proximal tail region, but they did not form the canonical 9 + 2 axoneme arrangement (Fig 8C). Microtubules were singlets and were not paired, and some seemed to be incomplete tubules (Fig 8C2 and 8E2). There was also evidence of uneven thickness in the tail staining with tubulin (GT335 and AcTu, Fig 4B), which coincides with the uneven microtubules presented in the ultrastructural images (Fig 6E and 6F). Nevertheless, most tubules were associated with electron-dense material that appears to be outer dense fibers and/or fibrous sheath (Fig 8C and 8E). The outer dense fibers and fibrous sheath were not properly organized and were scattered in various portions of the tail, though present, which is consistent with the reduced levels of ODF1 and AKAP4 markers (Fig 8E and 8F). The mitochondrial sheath contained centrally located mitochondria, but they were not properly organized (Fig 8F). Additionally, *Arl2bp* KO sperm at stage IX displayed manchette (Fig 8D2), and in sperm from testis sections, the basal plate and capitulum were present in the neck, but segmented columns were either absent or severely disrupted (Fig 8F2). We were unable to detect sperm at later stages in development after disassembly of the manchette and were therefore unable to fully assess manchette structure throughout spermatogenesis. Altogether, these data confirm the necessity for ARL2BP in sperm flagellum structure and formation.

### ARL2BP is necessary for left-right patterning in mice

**Fig 9:**
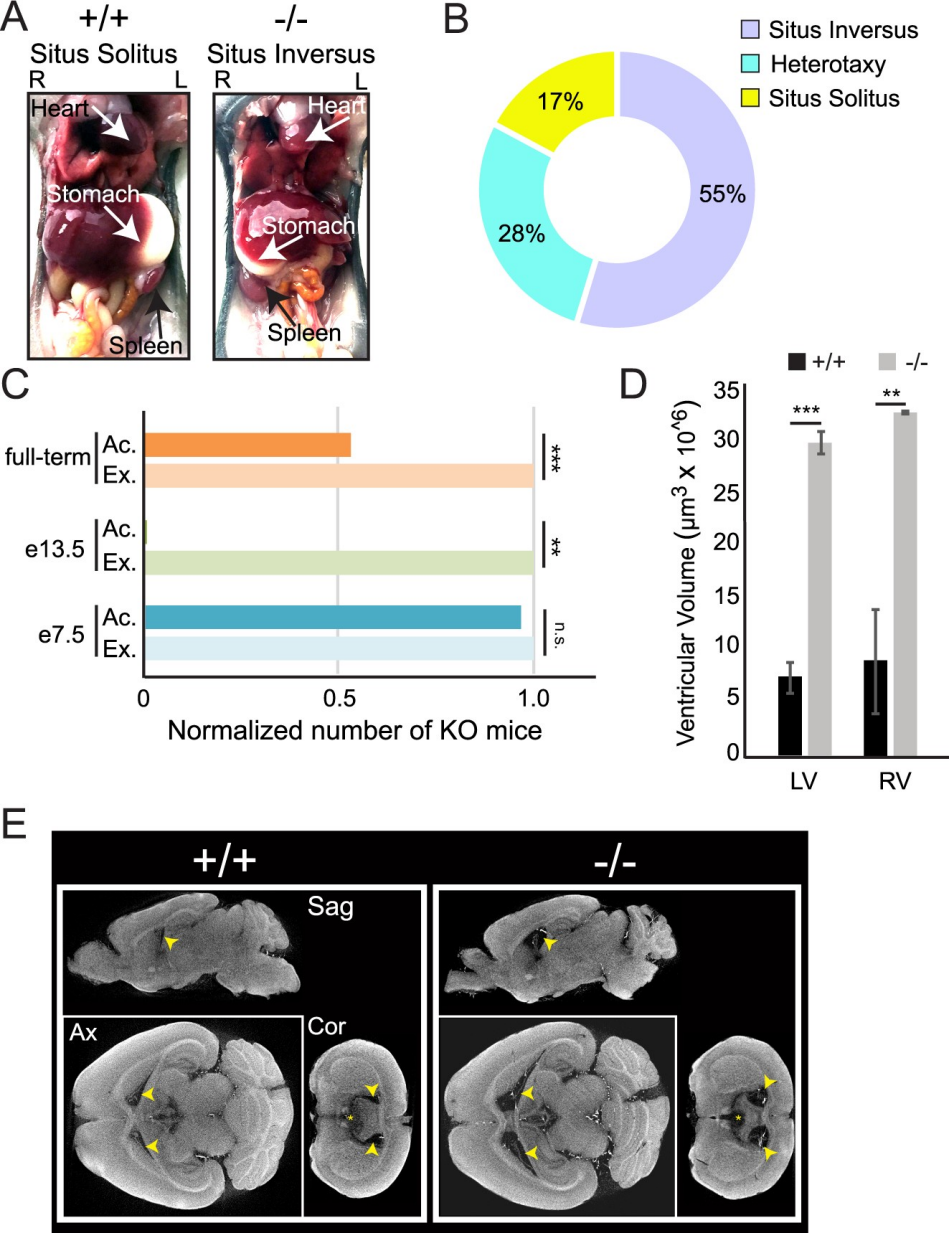
Laterality defects and enlarged lateral ventricles in the adult murine brain with absence of ARL2BP. (A) WT (+/+) mouse displaying situs solitus (normal axis patterning), and KO (-/-) mouse exhibiting situs inversus (complete reversal). L = Left and R = Right. (B) Percentages of KO animals with left/right-asymmetry abnormalities. (C) Graph displaying the non-Mendelian distribution of KO animals identified from Het x KO crosses of full-term litters (total of 100 animals with 23 KO’s, chi square value of 39.19, p>0.001), or collected at embryonic day 13.5 (e13.5) (total of 7 animals with 0 KO’s, chi square value of 7, 0.01>p>0.005), compared to the Mendelian distribution seen at embryonic day 7.5 (e7.5) (total of 24 animals with 16 KO’s, chi square value of 0.96). (D) Comparison of ventricular volumes between WT (+/+) and KO (-/-) brains from 3D-recontruction of CT images. Left p = 0.0002, Right p = 0.0087; according to unpaired, two-tailed *t* test (*n* = 3). Data are represented as the mean ± SEM. (E) Micro-CT scans of WT (+/+) and KO (-/-) murine brains at postnatal day 70, in Sagittal (Sag), Axial (Ax), and Coronal (Cor) planes. Arrows mark the lateral ventricles. Ac. = Actual #, Ex. = Expected #; LV = left ventricle, RV = right ventricle.

Human patients, as well as KO animals, were further examined for additional phenotypes typically observed in ciliopathies. The human patients in this study did not report any symptoms associated with impaired cilia in the trachea or kidney. This finding is corroborated by our observations in *Arl2bp* KO mice (S3, S4 and S5 Videos). However, we observed a high number of KO mice displaying situs inversus or heterotaxy, an asymmetric Left/Right (L/R) positioning of the internal organs caused by defects in the nodal cilia of developing embryos (Fig 9A). The association of *ARL2BP* mutations in humans with situs inversus is variable, and the patients reported in this study did not possess situs inversus. However, a previous study identified one patient with a mutation in *ARL2BP* with situs inversus [15]. In mice, this phenotype is highly penetrant, as most *Arl2bp* KO animals exhibit situs inversus (55%), with 28% possessing heterotaxy of either the heart or stomach (Fig 9B). Furthermore, tracking and statistical analysis revealed that the number of KO’s produced from Heterozygous x Knockout or from Heterozygous x Heterozygous parents did not follow Mendelian ratios when examined from full-term litters or from embryonic day 13.5 (e13.5) (chi square value of 39.19, p<0.001, and chi square value of 7, 0.01<p<0.005, respectively). However, litters examined from e7.5 did follow Mendelian ratios (chi square value of 0.96) suggesting that there may be partial embryonic lethality mediated by the absence of ARL2BP at or near the time of gastrulation (after day e7.5) (Fig 8C). Unfortunately, further studies delineating the timing of embryo loss was not in the scope of this study. Nonetheless, these results establish ARL2BP’s essential role in node-determined laterality during murine development.

### Cerebral ventricular volume affected with loss of ARL2BP

An additional ciliated tissue investigated in the *Arl2bp* KO mice was the cerebral ventricles. These are fluid-filled cavities in the brain that contain ciliated tufts on the apical surface of ependymal cells. There are four interconnected cavities, including the two lateral ventricles and third ventricle in the forebrain that are connected to the fourth ventricle in the brain stem by the cerebral aqueduct present in the midbrain. The motile cilia in these cavities are responsible for the circulation of cerebrospinal fluid (CSF) from the lateral ventricles, where it is produced, to the surrounding space of the brain and spinal cord. Disruption of the CSF flow results in enlarged ventricles and hydrocephaly due to build-up of CSF in the ventricles. The gross morphology of the rest of the brain appears similar to WT in size and shape. White matter axon tracts and gray matter volume appeared consistent between genotypes, indicating that deletion of *Arl2bp* does not result in gross structural brain malformations. Remarkably, a 4-fold increase in lateral ventricular volume was observed in the brains of KO mice compared to WT littermates using micro-CT scans (Fig 9D and 9E). Of note, the third ventricle also appeared enlarged in *Arl2bp* KO mice, however we were not able to accurately quantify this due to problems with segmentation from the surrounding space. Additionally, there was no noticeable difference in the cerebral aqueduct or fourth ventricle between WT and *Arl2bp* KO mice, nor was there any sign of obstruction in the cerebral aqueduct or fourth ventricle, a common cause of hydrocephaly. Interestingly, the majority of KO mice did not present external signs of hydrocephaly. Though the larger ventricular volume points to a role for ARL2BP in CSF flow regulation, a detailed behavioral analysis would need to be completed to understand if these structural changes cause any behavioral deficits.

To assess this phenotype in our patients, we looked for signs pointing to chronic hydrocephalus. None were shown nor reported by patients P1 and P3. On the other hand, patient P2 suffered from frequent migraines and recently underwent a cerebral CT imaging examination, which revealed normal ventricular volumes and absence of other morphological anomalies.

### Shortened primary cilia in the absence of ARL2BP

**Fig 10:**
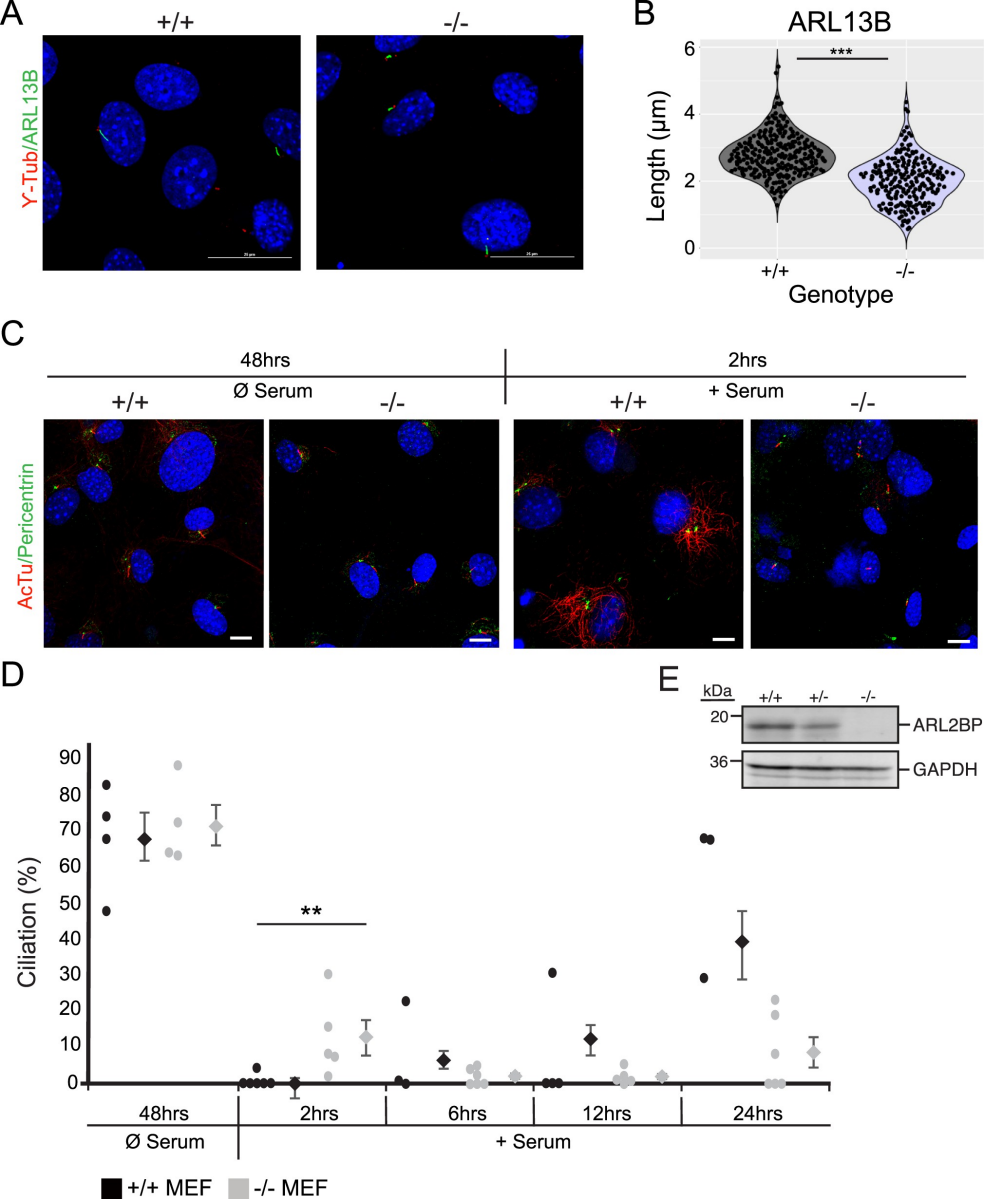
Cells lacking ARL2BP undergo slower ciliary depolymerization. (A) Mouse embryonic fibroblasts (MEFs) from WT (+/+) and KO (-/-) littermates stained to show DAPI (nuclei, blue), ARL13B (cilia, green), and γ-tubulin (basal body, red). (B) Violin plot displaying the length measurements from WT (+/+) and KO (-/-) MEFs. n = 200. P = 0.0001, according to unpaired, two-tailed *t* test (*n* = 200). (C) MEFs from WT (+/+) and KO (-/-) littermates after serum starvation (cilia induction) for 48 hours, followed by serum addition (cilia depolymerization) for 2 hours stained for Acetylated tubulin (AcTu–cilia, red) and pericentrin (basal body, green). (D) Dot plot displaying the percentage of ciliated cells between WT (+/+) and KO (-/-) MEFs after 48 hours of serum starvation and at indicated time points after re-addition of serum. Average percentage of ciliated cells is to the right of each dot set. Data are represented as the mean ± SEM. For 2hrs, p = 0.0174, other time points not statistically significant according to unpaired, two-tailed *t* test (*n* (each dot representing 200≥100 cells) = 3 or 4). (E) Immunoblot of WT (+/+), Heterozygous (+/-), and KO (-/-) MEF cell lysates probed for ARL2BP, with GAPDH as the loading control. Molecular weights in kilodaltons (kDa) are displayed on the left.

To further investigate the role for ARL2BP, mouse embryonic fibroblasts (MEFs) were generated from *Arl2bp* KO mice and WT littermates. Primary cilia were induced in MEFs after removal of serum from the growth media. After 48 hours of serum starvation, the percentage of ciliated cells was comparable between WT and KO (Fig 10C and 10D). MEFs lacking ARL2BP possessed significantly shorter cilia (average of 2μm) than WT MEFs (average of 2.7μm) (Fig 10A and 10B). Importantly, WT MEFs did express ARL2BP, whereas Arl2bp KO MEFs did not (Fig 10E). To determine if the cilia present in *Arl2bp* KO MEFs had difficulty in primary cilia depolymerization or re-entry into the cell cycle, cilia resorption was assessed after addition of serum to cilia-induced cells. Interestingly, after 2 hours of serum addition, a significantly higher percentage of KO MEFs retained their cilia than what was observed in WT (Fig 10C and 10D). Further observation of KO and WT MEFs revealed that cilia resorption was not statistically different between them from 6 to 24 hours (Fig 10D). Importantly, cell cycle distribution in KO MEFs was similar to that of WT, as evaluated through cell sorting and observed throughout primary MEF cell maintenance (S2 Table), thus the observed effects on primary cilia statistics are cell cycle independent. These results indicate that loss of ARL2BP affects the initial depolymerization of primary cilia.

## Discussion

In this study, we sought to understand the mechanism behind ARL2BP dysfunction by examining both patients and mice. With our findings, we validate the past association with the blinding disease RP, and bring the first evidence of ARL2BP involvement in spermatogenesis. We show that additional cilia-related phenotypes originate from the loss of full- length ARL2BP, which point to the need for ARL2BP in cilia and flagella structure and maintenance.

Since its discovery, the function of ARL2BP has remained largely elusive. Initial characterization was established by its interaction with ARL2, a member of the ARF-like (ADP ribosylation factor) family which are a group of small GTP-binding proteins belonging to the RAS superfamily [23–25]. A recent report using constitutively active ARL2 (Q70L mutation) in retina demonstrates a defect in outer segment morphology with rapid loss of photoreceptor function [26]. However, *Arl2bp* KO does not phenocopy the constitutively active *Arl2* murine model, and in addition, our attempts to show this interaction in sperm or retina have been unfruitful (16). Past publications have established that dysfunctional ARL2BP impacts the photosensory cilium in humans and mice [12, 16]. Experiments in human retinal pigment epithelial cells showed that *ARL2BP* knockdown with small interfering RNA caused reduced cilia length [12], and similarly, germline deletion of *Arl2bp* in mice showed shortened photoreceptor cilia with impairment of ciliary doublet microtubule structure (open inner junction) [16], as corroborated by our data from MEFs.

### ARL2BP is essential for sperm flagellar midpiece formation in humans and mice

Spermatogenesis begins in the basal compartment at the outer edge of the seminiferous tubules and continues inward until fully formed spermatids are released into the lumen and on to the epididymal tissues. The last stage, spermiogenesis, is characterized by sperm tail formation, beginning with axoneme growth and assisted by a transient microtubular structure, the manchette [6].

Without ARL2BP, sperm tails fail to elongate. Morphological analysis with Transmission Electron Microscopy (TEM) revealed that the 9+2 axoneme structure was disorganized, with no detectable doublet microtubules (DMT). The outer dense fibers and fibrous sheath structures still accumulate around the tail during spermatogenesis but fail to assemble appropriately. This coincides with the loss of ODF1 (ODF) and AKAP4 (FS) staining, and the increase of residual bodies present in testes of KO animals, an indicator of failed spermiogenesis. Furthermore, the increased levels of phosphorylated AKAP4 in the testes instead of the sperm of KO mice points to the inability to properly assemble the FS present throughout the principal piece. The presence of p-AKAP4 in the testes lysates is likely related to the fibrous sheath fragments seen in the cytoplasmic bulges present in electron micrograph images of the testes, as well as the AKAP4-containing residual bodies. The misassembled FS is also linked to the irregular axoneme structure, as the ODFs attach directly to their corresponding DMTs in the principal piece [27, 28]. FS assembly occurs in a distal to proximal direction, following formation of the axoneme. Without the stable interaction and growth of the DMTs and ODFs there is not creation of a true “distal end”. Likely, the smaller, disorganized clumps that show FS periodicity and rib-like appearance in the ultrastructural images is due to the inability of the FS precursor to assemble correctly along the distal axoneme. Furthermore, AKAP4 processing and FS assembly is dependent on the formation and proper localization of the annulus. This is a septin ring-structure formed during spermiogenesis that travels to its position between the mid- and principal pieces after midpiece formation is complete [29] (S2 Fig). If the midpiece forms improperly, the annulus cannot localize appropriately, resulting in abnormal processing of AKAP4 and a failure to complete spermiogenesis.

These defects in FS, and thereby principal piece formation, are accompanied by issues in midpiece formation. In the midpiece, the ODF does not directly bind to the microtubule doublets, but it is held in place by, and in early spermatogenesis attached to, the surrounding mitochondrial sheath (MS) [30, 31]. The lack of a properly assembled MS could be attributed to the poorly assembled ODFs. Therefore, it is possible that without ARL2BP, the malformation in the microtubule structure causes impaired assembly of all periaxonemal structures and a failure to complete spermiogenesis. This is evident by the lack of principal pieces (which assemble last) in sperm tails from the KO model and in the majority of sperms from the human sample.

### Putative role for ARL2BP in intra-manchette transport (IMT)

The manchette is a transient microtubule- and F-actin-based structure formed during spermiogenesis. The formation and assembly of the MS, ODF, FS, and acrosome is mediated by intra manchette transport (IMT), a process resembling intraflagellar transport (IFT) [29, 32, 33]. Given that the MS, ODF, FS and acrosome assembly depends on ARL2BP, and that the manchette forms in *Arl2bp* KO sperm, we hypothesize that IMT is impaired or that there is a delay in the manchette disassembly. This hypothesis is supported by animal models that lack SPEF2, a protein required for the axonemal central pair in sperm flagellum, and MEIG1, a protein required for spermiogenesis. Loss of either of these proteins results in abnormal head and tail formation, which in both cases are linked to an elongated or delayed manchette [34–39]. Though it is unlikely that ARL2BP directly binds with SPEF2 or MEIG1, the abnormally shaped heads and impaired assembly of accessory structures with loss of these proteins is similar, indicating that ARL2BP may be involved in IMT or manchette disassembly. Furthermore, we showed ARL2BP localizing in the equatorial zone of the acrosome, as well as abnormal acrosome formation in the *Arl2bp* KO sperm. In mammals, proteins localizing at the equatorial segment of the acrosome are involved in the initiation of sperm-oocyte fusion [40, 41]. However, it remains unclear if ARL2BP has any direct role in fertilization. Taken together with the data observed from the WT and *Arl2bp* KO MEFs, the delay in cilia depolymerization closely relates to the notion that ARL2BP is involved in manchette disassembly. Therefore, we hypothesize that ARL2BP is essential for depolymerization of microtubules in the manchette and MEF primary cilium.

We cannot, however, exclude the possibility that lack of ARL2BP in Sertoli cells underlies part, or all, of the phenotypes observed in sperm from patients and *Arl2bp* KO mice. Considering that Sertoli cells are essential to the regulation of spermatogenesis, and are located directly next to sperm cells throughout their developmental lifetime, it is possible that loss of ARL2BP compromises the ability of these cells to nourish the sperm cells or take up the residual bodies.

### ARL2BP is involved in organ laterality

L/R-asymmetry is determined by the mono-ciliated cells in the embryonic node during gastrulation. There is a leftward-fluid flow generated in this region by motile monocilia, which bend the immotile cilium of the crown cells and signals for asymmetrical protein expression related to eventual organ placement [42, 43]. Therefore, if this fluid flow is disrupted, the laterality of the organs will be affected. It was shown in an elegant study by Nonaka, *et*. *al*. 2002 that complete reversal of the flow (rightward) resulted in complete situs inversus [44]. However, what determines situs inversus vs other forms of laterality placements are not known and is thought to be stochastic. To date, one patient with defective ARL2BP was reported to have situs inversus [12]. This laterality defect was also observed in KO mice, but not in patients from the two families of this study, who have normal organ position (situs solitus). However, there was an abnormally high incidence (55%) of situs inversus, compared with normal laterality or heterotaxia in the KO mice. Since the axonemes in photoreceptors and sperm tails are shorter with loss of ARL2BP, it is likely that nodal cilia are also shorter in the KO animal. Interestingly, throughout the breeding process, we noticed a significant decrease in the expected number of KO animals after laterality had been determined (e7.5). Therefore, we hypothesize that some KO embryos possessing heterotaxia died embryonically. Since different forms of heterotaxia are related to embryonic lethality (heart malformations, congenital heart disease), this could be possible [45, 46]. This could also explain why only one patient identified with *ARL2BP* mutations possesses situs inversus, while the incidence in the mouse model is much higher. Though these questions could not be answered within the scope of this study, these results provide an interesting avenue for further research.

### ARL2BP is not essential for the function of multi-ciliated cells

Multi-ciliated cells are present in the ventricles of the brain, throughout the trachea, and in the fallopian tubes. Patients with mutations in *ARL2BP* were not reported to have any symptoms related to defects in these cilia (sinusitis, otitis media, hydrocephaly, or female infertility). Among patients of this study, only P4 was reported with chronic bronchitis. The late age of onset of this respiratory issue and the fact that the patient is presently deceased, makes it difficult to discern possible association of this to mutated *ARL2BP*-driven ciliary defects. To note, the mouse model also did not display any related symptoms. Interestingly however, live imaging of dissected trachea of KO mice possessed tufts of normal cilia, immotile cilia, and cilia with uncoordinated beating (S3, S4 and S5 Videos) [47]. Though overall, the tracheal cilia from the KO mice were able to move fluid in one direction, similar to WT. Likewise, the ventricular volumes in KO mouse brains were significantly increased but neither of these defects were enough to cause a discernible phenotype. Therefore, we consider that ARL2BP is not required for the function of multi-ciliated cells, though it does appear to be necessary for the robust performance of these cilia.

### Conclusions

With our findings, we validate the past association of *ARL2BP* mutations with the blinding disease RP, and bring the first evidences of ARL2BP involvement in spermatogenesis. We thereby prove that additional cilia-related phenotypes originate from ARL2BP deficiency, with manifestations that are similar in human and mouse. Furthermore, we provide a first insight into the disease mechanisms associated with *ARL2BP* mutations in relationship to defective ciliogenesis, pointing to an essential role for this protein in the maintenance of normal structure and homeostasis of cilia and flagella.

## Materials and methods

### Ethics statement

Protocol # 09/14 approved by the Cantonal Committee (Vaud Canton) for Research Activities on Human Subjects, Title: "Molecular Genetics of Ocular Diseases"—written consent was given. On 3/15/2017 Comissão de Ética para a Saúde do Instituto de Oftalmologia Dr. Gama Pinto approved research on human patients. Written consent was given. On 4/28/2017 Comissão de Ética para a Saúde do Instituto de Oftalmologia Dr. Gama Pinto approved a few amendments to the human patient protocol. Written consent was given. This study followed the Guide for the Care and Use of Laboratory Animals of the National Institutes of Health. All animals used in this study were handled and housed according to approved Institutional Animal Care and Use Committee (IACUC) protocol # 1803013440 of West Virginia University. The approved euthanasia procedure used was carbon dioxide inhalation followed by cervical dislocation.

### Patients and controls

Patients were recruited from the Instituto de Oftalmologia Dr. Gama Pinto in Lisbon, Portugal. DNA was extracted from peripheral blood leukocytes (subjects P1 and P2), and from saliva (subject P3), after obtaining written informed consent. RNA was extracted from a sperm sample from patient P1. A control sperm and DNA sample were provided by a healthy donor.

### Clinical evaluation

Ophthalmologic examination included assessment of BCVA, slit-lamp examination, dilated fundus examination, fundus photography, visual fields, and optical coherence tomography (OCT). Full‐field ERGs were also recorded, following the International Society for Clinical Electrophysiology of Vision (ISCEV) protocol. Semen analysis (subject P1) was carried out by standard procedures by an andrology laboratory, and according to WHO guidelines [17]. Additional clinical features were assessed based on patients’ clinical history.

### Whole-exome sequencing (WES)

WES was performed using 2 μg DNA derived from peripheral blood mononuclear cells. Protein-coding regions were captured using the HiSeq Rapid PE Cluster Kit v2, and an Illumina HiSeq 2500 instrument was used for paired-end sequencing. Single nucleotide variants and small insertions and deletions were detected using the Genome Analysis Tool Kit (GATK v4.0) software package, using the Best Practice Guidelines identified by the developers [48]. The pathogenicity of the detected genetic variants was assessed after functional annotation through ANNOVAR [49] and with the mean of in-house scripts [50]. Genomic regions with high homozygosity were determined using the AutoMap software.

### Sanger sequencing validation, PCR, and RT-PCR

Primer pairs for *ARL2BP* exons and flanking intron boundaries were designed using the CLCbio Genomics Workbench (Qiagen). PCR amplification was performed in a 10 μl total volume containing 2 ng genomic DNA or cDNA, 1x GoTaq buffer, 0.1 mM dNTPs, 10 μM of each primer, and 5 U/μl of GoTaq polymerase (Promega). PCR products were purified using ExoSAP-IT, USB, or extracted from agarose gel using Nucleospin Gel and PCR Clean-up (Macherey-Nagel). Sanger sequencing was performed by a service provider (Fasteris, http://www.fasteris.com/). Reverse transcription was performed on 1 μg RNA using a mix of random primers and the GoScript Reverse Transcription Protocol (Promega).

### Immunoblotting

Mice were euthanized by CO_2_ inhalation and testes were dissected and separated from epididymis and fat. Testis samples were weighed and immediately frozen in liquid nitrogen. Prior to analyses, the samples were homogenized in phosphate buffered saline (PBS, with protease inhibitor cocktail (Thermo Fisher #A32955)) and a NanoDrop spectrophotometer was used to measure protein concentrations. Samples were analyzed by SDS-PAGE gel, followed by transfer onto polyvinylidene difluoride membranes. After blocking the membranes with western blot blocking buffer (LiCor 004864 Classic) for 30 min at room temperature, they were incubated with the primary antibodies overnight at 4°C. The membranes were then washed in PBST (PBS with 0.1% Tween-20) 3 times for 5 minutes (3 X 5 min) at room temperature before incubation in secondary antibody (goat anti-rabbit Alexa 680, goat anti-rat Alexa 680, or goat anti-mouse Alexa 800) for 30 min at room temperature. After 3 x 5 minutes of washes with PBST, membranes were scanned using Odyssey Infrared Imaging System.

### Immunofluorescence

For testis, the anesthetized animal was perfusion fixed with 4% PFA and then the testis was dissected out. This was followed by incubation in 30% sucrose/PBS overnight at 4°C. Afterward, testes were incubated in a 1:1 mixture of 30% sucrose in PBS and OCT (Cryo Optimal Cutting Temperature Compound, Sakura) for 1 hr, and flash frozen in OCT. Staining was performed following the same protocol as detailed below. For sperm, the animal was euthanized by CO_2_ inhalation, and the epididymis was collected. The tissue was then minced, and sperm were allowed to swim out for 5–10 minutes (in the case of knockout, entire volume of liquid was collected) and collected into an Eppendorf tube. 100μl of sperm suspension was added to a Superfrost Plus slide and allowed to completely dry by placing the slides on a hot plate (setting 3, Corning Hot Plate). Cells were fixed in 4% PFA for 15 minutes followed by three 5 minute washes in PBS. Ice-cold methanol was then added for 2 minutes, followed by three 5 minute washes in PBS. The cells were then blocked overnight at 4°C (PBS with 5% goat sera, 0.5% TritonX-100, 0.05% sodium azide). The next day, the cells were incubated with primary antibody for 2 hours at RT, followed by three 5 minute washes in PBS. The cells were then incubated with secondary antibody for 1 hour, followed by three 5 minute washes in PBS. They were then mounted with ProLong Gold anti-fade reagent and viewed on a Nikon confocal microscope.

All of the immunofluorescence performed on testes tissue and epididymal sperm was performed on at least 3 KO animals with corresponding littermate controls. Additionally, at least 2 fields of view were taken for each stain with each animal and representative images were selected.

Immunofluorescence for human P1 and control sperm samples was performed as described in previously-published protocols [7, 51]. The same anti-ARL2BP primary antibody described above was used in the human sperm staining experiments, in addition to a secondary goat anti-rabbit antibody conjugated with Alexa Fluor 488 (Invitrogen). Pictures were taken on a Zeiss LSM 780 confocal microscope.

### Cell counting

Mice were euthanized by CO_2_ inhalation and epididymal sperm were dissected out and placed in 1ml PBS. Sperm were collected, spun at 1500 RPM for 3 minutes and re-suspended in 50μl of PBS (knockout) or 4% paraformaldehyde (wild type; and further diluted 1:10 in PBS after 5 min). Sperm cells were counted using a hemocytometer.

### MEF cells

Embryos were harvested at E13.5 and separated from each other and the placenta in PBS. The tissues were minced with sterile razor blades. The tissues were then trypsinized with 6ml total of 0.25% Trypsin/EDTA. 7ml of MEF media (catalog # 10-013-CV from Corning) DMEM containing glucose, pyruvate, and L-glut + 15%FBS, 1% Pen/Strep) was then added to each tube and spun down at 1200 RPM for 8 minutes. Cells were then added to a 100mm dish containing 25ml of MEF media and allowed to grow overnight at 37°C with 5% CO_2_. Cells were maintained for up to 4 passages. To induce cilia formation, cells were grown to 90% confluency and serum starved for 48 hours. Depolymerization of cilia was done by the addition of sera (after 48 hours of starvation), and cells were collected for staining after 2 hours, 6 hours, 12 hours, or 24 hours of serum addition.

For immunocytochemistry, cells were fixed with 4% PFA for 15 min (or -20°C Methanol for 2 minutes for centrosomal staining, acetylated tubulin and pericentrin), washed with PBS 3 x 5 minutes, and blocked for 30–60 minutes (PBS with 5% goat sera, 0.5% TritonX-100, 0.05% sodium azide). The remaining steps were performed in the same way as outlined in the immunofluorescence section.

### Transmission electron microscopy

Mice were euthanized by CO_2_ asphyxiation and testes were dissected and separated from epididymis and fat. Testes were decapsulated and 1-3mm pieces were placed into 4% paraformaldehyde and 2% glutaraldehyde in 0.2M cacodylate buffer overnight at 4°C. Sperm were also collected from epididymal tissues and placed in the same fixative in the same conditions. Testis and sperm samples were then post-fixed in 1% osmium tetroxide, dehydrated in a series of increasing ethanol concentration, and embedded in Embed812 resin (Electron Microscopy Sciences, Hatfield, PA). Thin sections were cut and stained with UranyLess (EMS, Hatfield, PA) and photographed on a Hitachi HT7700 TEM (NSF grant #1229184). TEM analysis was performed on two WT mice and two KO mice.

### Micro-CT scans

Transcardial perfusions were performed on anesthetized 60 day old mice with 4% paraformaldehyde. The brain was carefully dissected out of the skull and fixed in 4% paraformaldehyde for 2 days at 4°C. The brains were then transferred to stability buffer (4% w/v paraformaldehyde (pH 7.2), 4% w/v acrylamide, 0.05% w/v bis-acrylamide, 0.25% w/v VA044 initiator, 0.05% w/v Saponin, in 1xPBS) for 3 days at 4°C, and then underwent nitrogen desiccation, followed by a 3 hour incubation at 37°C. After a two-day staining in 0.1N iodine, the brains were embedded into 3% agarose for imaging. The brains were imaged on a Bruker SkyScan 1272 MicroCT scanner (Cu 0.11μm filter, 1500ms exposure, and 8μm resolution), and 3D reconstruction of ventricular volume was performed with Seg3D software.

### Statistics

All data are presented as mean ± standard error margin. Immunoblots and ciliated cell counts were analyzed by unpaired, two-tailed *t* test (*n* = 3). For cilia measurements, staining was performed in triplicate with 100 cilia measured for each and data were visualized with the ggplot2 package in R version 3.3.2. Image and densitometry analysis were performed using ImageJ 1.50i. For ciliated cell counting, staining was performed in triplicate with 100 cells (and their cilia) counted for each. Mendelian ratios were analyzed using a chi square test with 1 degree of freedom. Chi square values of 3.84 or higher are statistically significant (p>0.05).

### Study approval

Our research has been conducted in accordance with the tenets of the Declaration of Helsinki and was approved by the Institutional Review Boards of our respective Organizations. Protocol # 09/14 was approved by the Cantonal Committee (Vaud Canton) for Research Activities on Human Subjects, Title: "Molecular Genetics of Ocular Diseases". On 3/15/2017 Comissão de Ética para a Saúde do Instituto de Oftalmologia Dr. Gama Pinto approved research on human patients. On 4/28/2017 Comissão de Ética para a Saúde do Instituto de Oftalmologia Dr. Gama Pinto approved amendments to the human patient protocol. Written informed consent was obtained from all patients prior to the sample collection. This study followed the Guide for the Care and Use of Laboratory Animals of the National Institutes of Health. All animals used in this study were handled and housed according to the approved Institutional Animal Care and Use Committee (IACUC) protocol #1803013440 of West Virginia University. The approved euthanasia procedure used was carbon dioxide inhalation followed by cervical dislocation.

## Supporting information

S1 Fig Visual electrophysiology exam of patient P1 (ID: LL1). Electroretinogram recordings from patient P1. LE = left eye, RE = right eye.
(JPG)

S2 Fig Scheme of WT and KO sperm structure. The fully developed sperm tail consists of four regions, including the connecting piece, the mid piece, the principal piece, and the end piece **(A)**. The additional structures associated with the axoneme differ throughout these segments, with a mitochondrial sheath (MS) surrounding the mid piece, and a fibrous sheath (FS) surrounding the principal piece. These sheaths surround 9 outer dense fibers (ODFs) that correspond to the 9 doublets of the axoneme, except when ODFs 3 and 8 are replaced by 2 longitudinal columns of the fibrous sheath. Lastly, these surrounding structures are shed in the end piece, which consists of just the microtubule axoneme **(A and B)**. In mature ARL2BP KO sperm, the FS and ODFs are lost, consisting only of an abnormally arranged MS and impaired flagellar axoneme **(C)**.
(TIF)

S1 Table Genes analyzed in patient P1. *P = Present.
(XLSX)

S2 Table Cell-cycle analysis of WT and *Arl2bp* KO MEFs. MEF cells were grown to confluency, fixed in ethanol, stained with propidium iodide. Flow cytometry was performed and there was no statistical difference in cell-cycle distribution between WT and KO MEF cells.
(DOCX)

S1 Video Wild-type murine sperm. (AVI)

S2 Video *Arl2bp* KO murine sperm. (AVI)

S3 Video Wild-type murine tracheal cilia. Trachea were dissected out according to published protocol [47] and placed on a glass-bottom dish, filled 1X PBS containing microbeads. The cilia are seen to beat and move the beads in one direction.
(AVI)

S4 Video *Arl2bp* KO murine tracheal cilia. Trachea were dissected out according to published protocol [47] and placed on a glass-bottom dish, filled 1X PBS containing microbeads. Some tufts of cilia show uncoordinated beating, but the beads are moved in one direction.
(AVI)

S5 Video *Arl2bp* KO murine tracheal cilia. Trachea were dissected out according to published protocol [47] and placed on a glass-bottom dish, filled 1X PBS containing microbeads. Some cilia tufts are immotile, but the beads are moved in one direction.
(AVI)

S1 File Uncropped immunoblot gels. (PPTX)
