# Supplementary figures and images for: Mutations in ARL2BP, a protein required for ciliary microtubule structure, cause syndromic male infertility in humans and mice
Source: PLoS Genet. 2019 Aug 19;15(8):e1008315. doi: 10.1371/journal.pgen.1008315 (PMC6715254; doi:10.1371/journal.pgen.1008315)

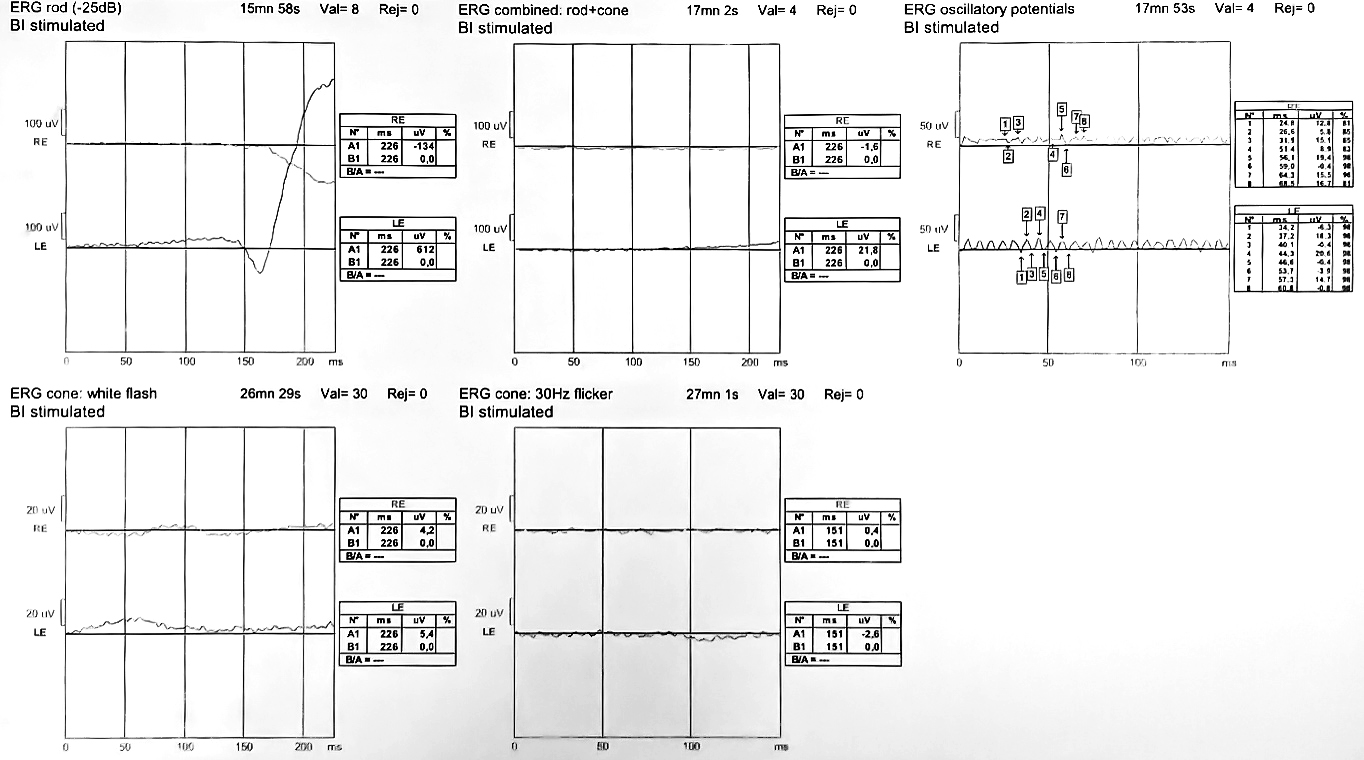

Supplement: S1 Fig — Electroretinogram recordings from patient P1. LE = left eye, RE = right eye. (JPG) [file pgen.1008315.s001.jpg]

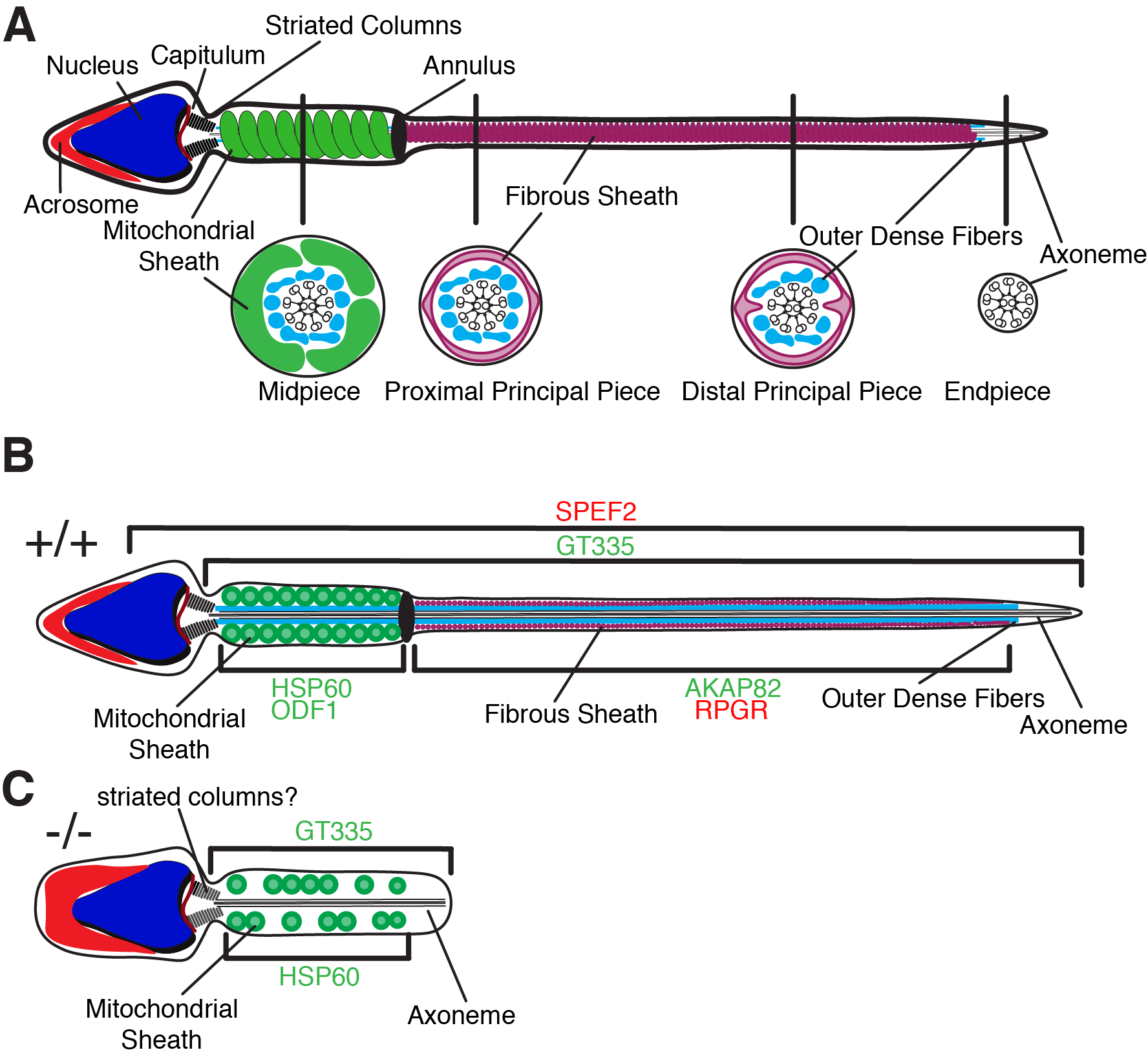

Supplement: S2 Fig — The fully developed sperm tail consists of four regions, including the connecting piece, the mid piece, the principal piece, and the end piece (A). The additional structures associated with the axoneme differ throughout these segments, with a mitochondrial sheath (MS) surrounding the mid piece, and a fibrous sheath (FS) surrounding the principal piece. These sheaths surround 9 outer dense fibers (ODFs) that correspond to the 9 doublets of the axoneme, except when ODFs 3 and 8 are replaced by 2 longitudinal columns of the fibrous sheath. Lastly, these surrounding structures are shed in the end piece, which consists of just the microtubule axoneme (A and B). In mature ARL2BP KO sperm, the FS and ODFs are lost, consisting only of an abnormally arranged MS and impaired flagellar axoneme (C). (TIF) [file pgen.1008315.s002.tif]
