# Supplementary material for: Mutations in ARL2BP, a protein required for ciliary microtubule structure, cause syndromic male infertility in humans and mice
Source: PLoS Genet. 2019 Aug 19;15(8):e1008315. doi: 10.1371/journal.pgen.1008315 (PMC6715254; doi:10.1371/journal.pgen.1008315)
Supplement: S2 Table — MEF cells were grown to confluency, fixed in ethanol, stained with propidium iodide. Flow cytometry was performed and there was no statistical difference in cell-cycle distribution between WT and KO MEF cells. (DOCX) [file pgen.1008315.s004.docx]

| **WT MEF** | **Passage** | **G1, %** | **G2, %** | **S, %** |
| --- | --- | --- | --- | --- |
| WT MEF | P0 | 77.62 | 11.02 | 11.36 |
| WT MEF | P1 | 82.37 | 12.89 | 4.74 |
| WT MEF | P2 | 84.42 | 5.63 | 9.95 |
| **Mean ± SD** |  | **81.47 ± 3.49** | **9.85 ± 3.77** | **8.68 ± 3.49** |

| **ARL2BP KO MEF** | **Passage** | **G1, %** | **G2, %** | **S, %** |
| --- | --- | --- | --- | --- |
| ARL2BP KO MEF | P2 | 79.64 | 15.52 | 4.85 |
| ARL2BP KO MEF | P2_2 | 79.75 | 12.14 | 8.11 |
| ARL2BP KO MEF | P3 | 87.67 | 9.97 | 2.36 |
| ARL2BP KO MEF | P4 | 77.51 | 18.2 | 4.3 |
| ARL2BP KO MEF | P4_2 | 79.64 | 16.01 | 4.35 |
| **Mean ± SD** |  | **80.84 ± 3.93** | **14.37 ± 3.28** | **4.79 ± 2.08** |
