# Supplementary material for: Mutations in ARL2BP, a protein required for ciliary microtubule structure, cause syndromic male infertility in humans and mice
Source: PLoS Genet. 2019 Aug 19;15(8):e1008315. doi: 10.1371/journal.pgen.1008315 (PMC6715254; doi:10.1371/journal.pgen.1008315)
Supplement: S1 File — (PPTX) [file pgen.1008315.s010.pptx]

## Slide 1
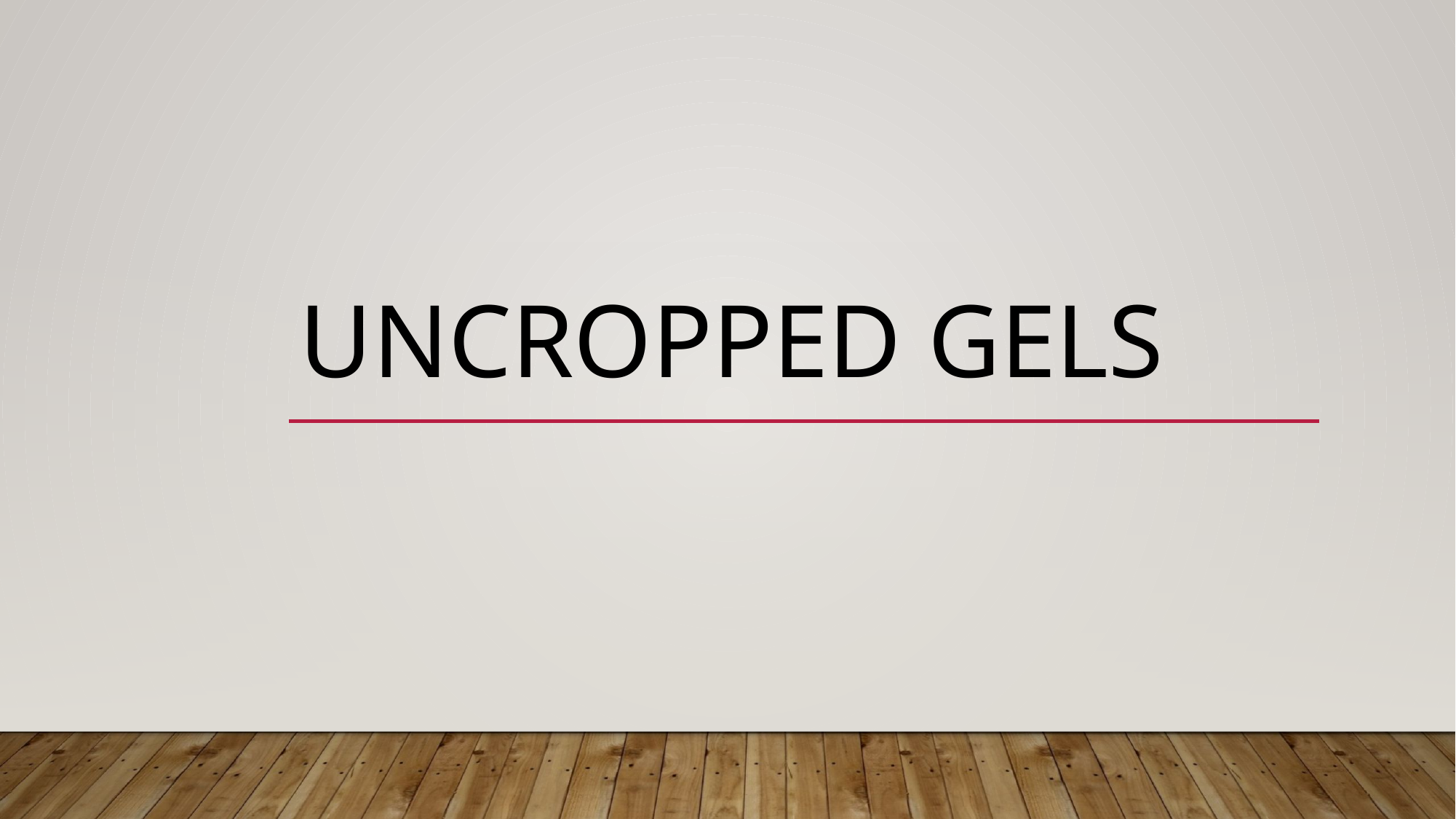

# Uncropped gels

## Slide 2
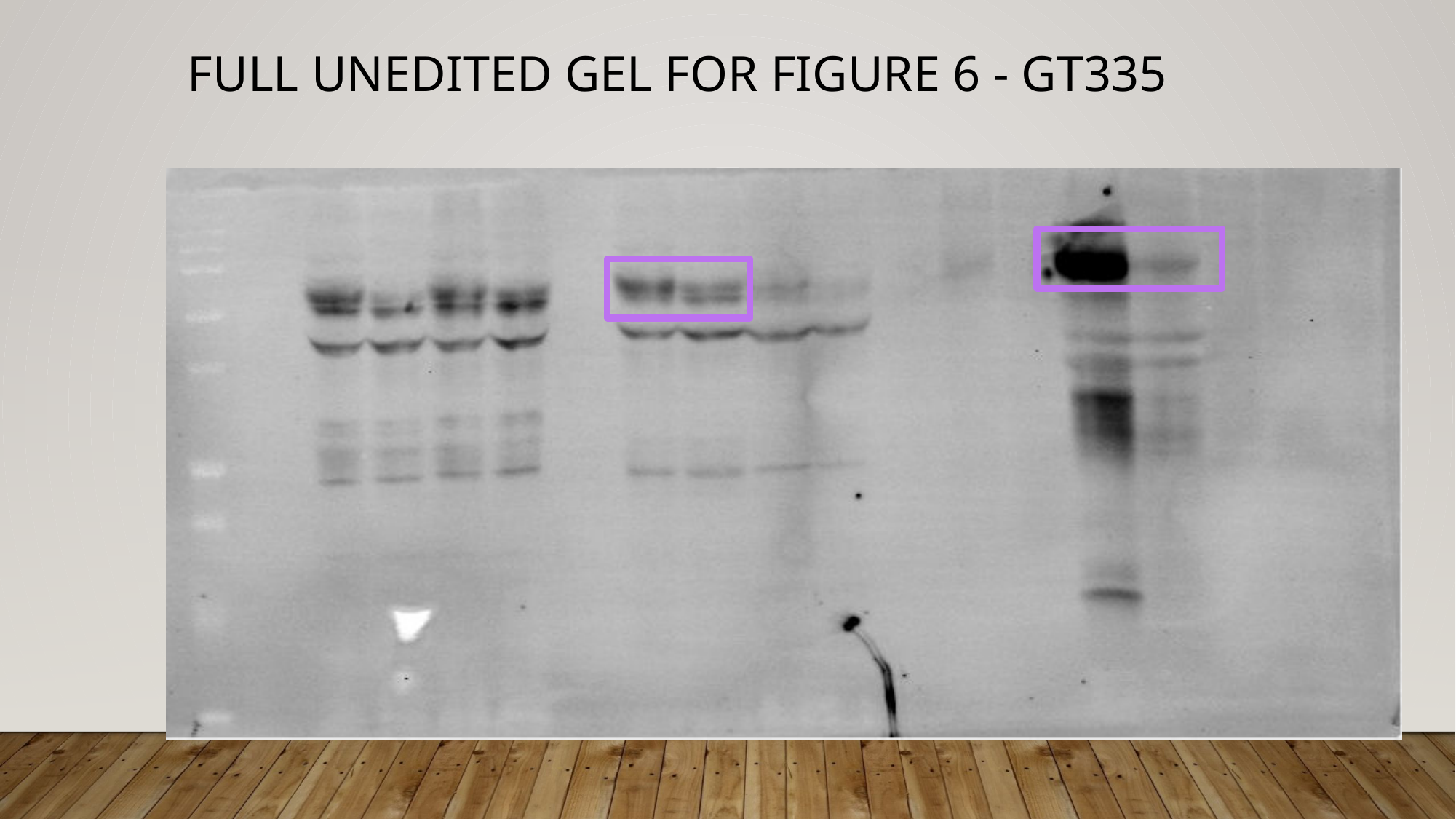

# Full unedited gel for Figure 6 - GT335

## Slide 3
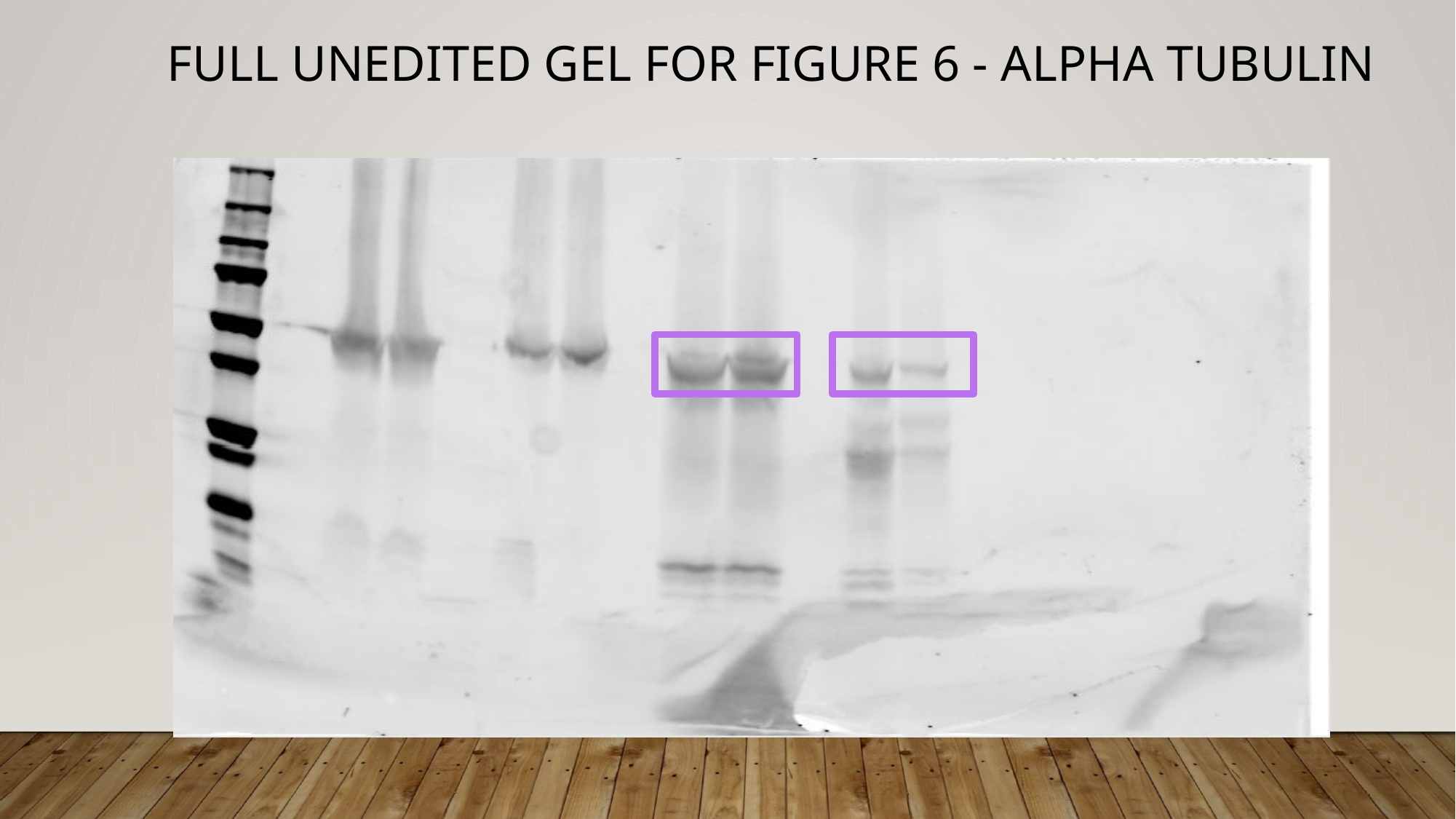

# Full unedited gel for Figure 6 - Alpha tubulin

## Slide 4
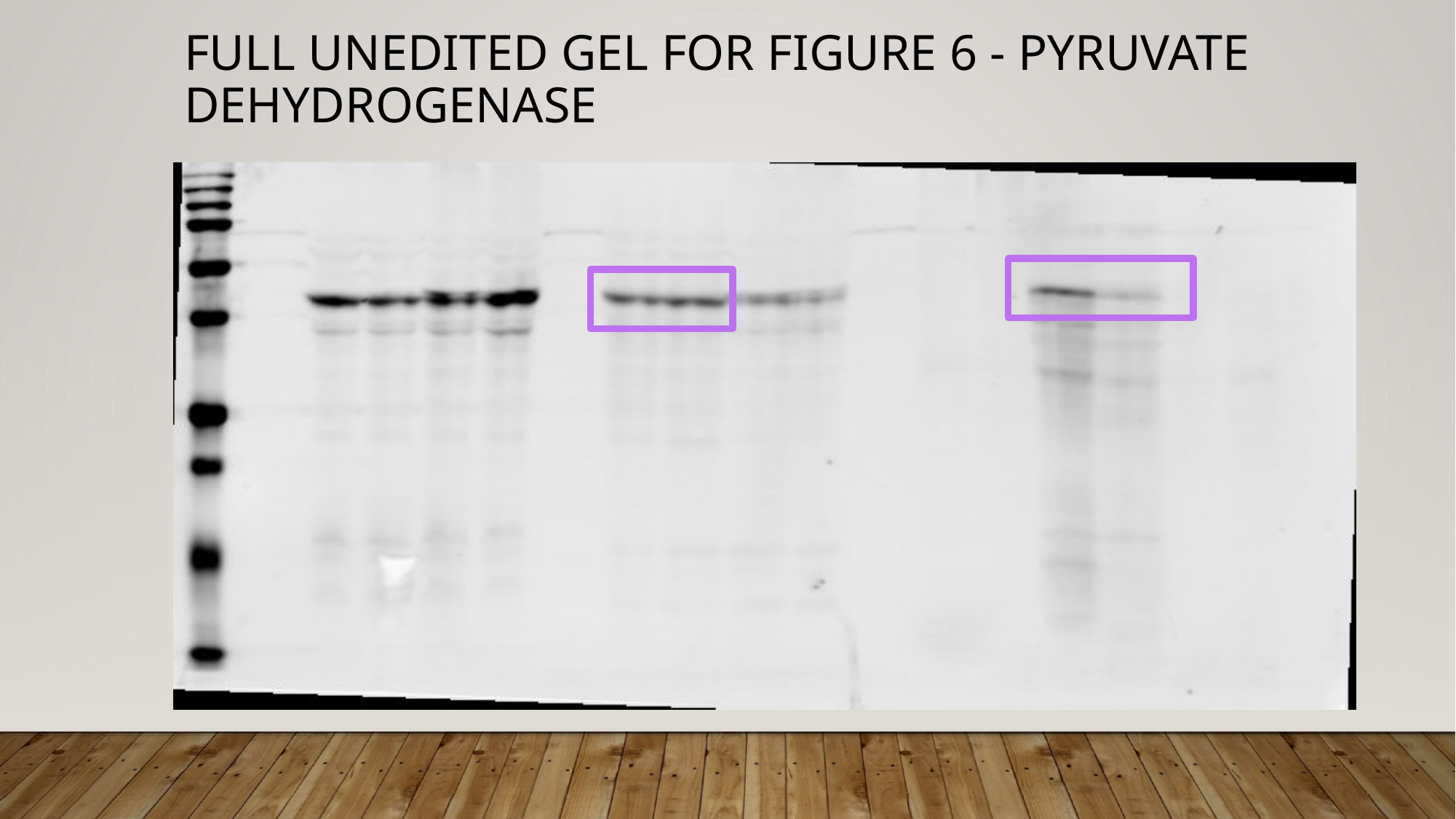

# Full unedited gel for Figure 6 - Pyruvate Dehydrogenase

## Slide 5
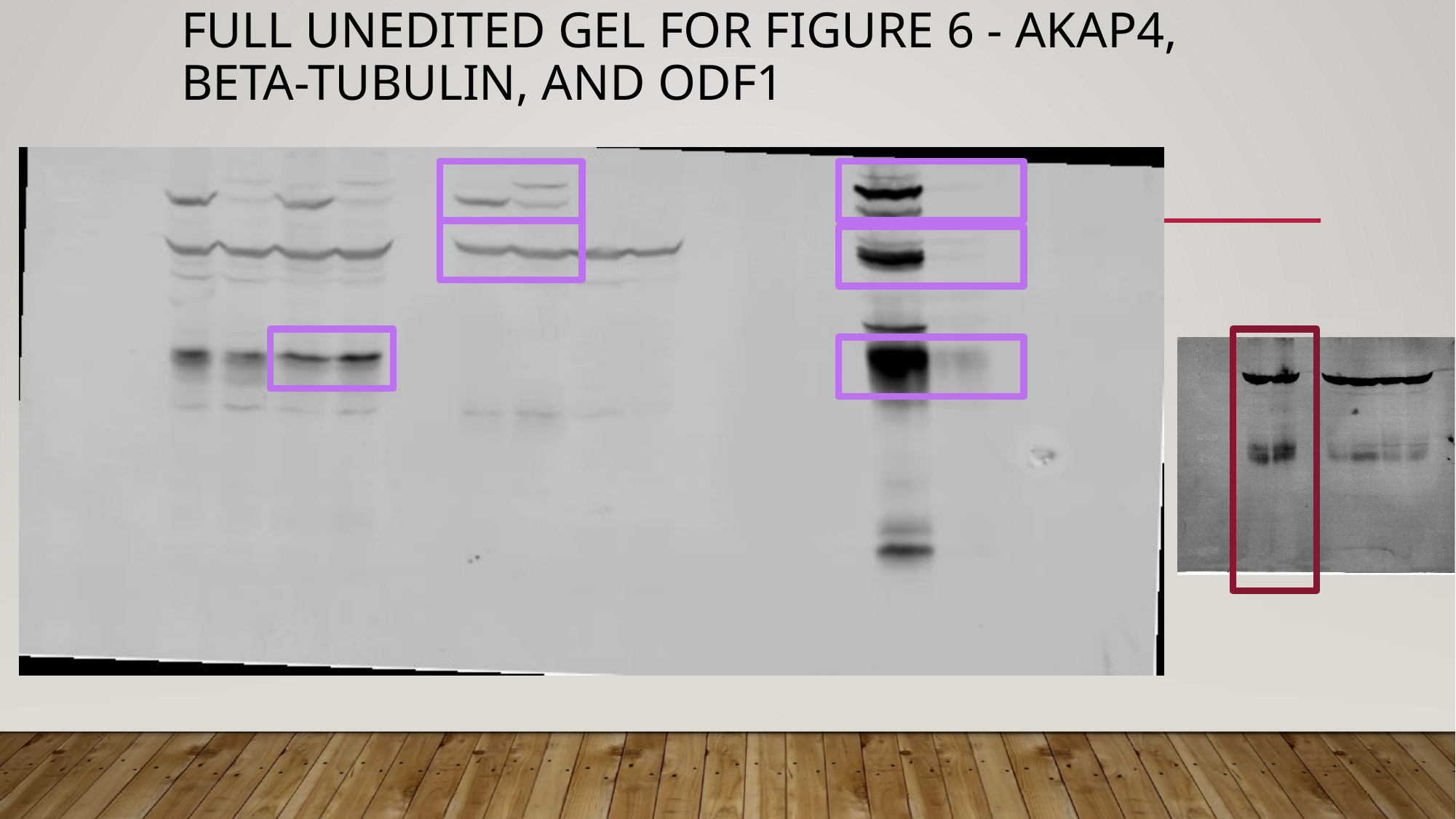

# Full unedited gel for Figure 6 - AKAP4, beta-tubulin, and ODF1

## Slide 6
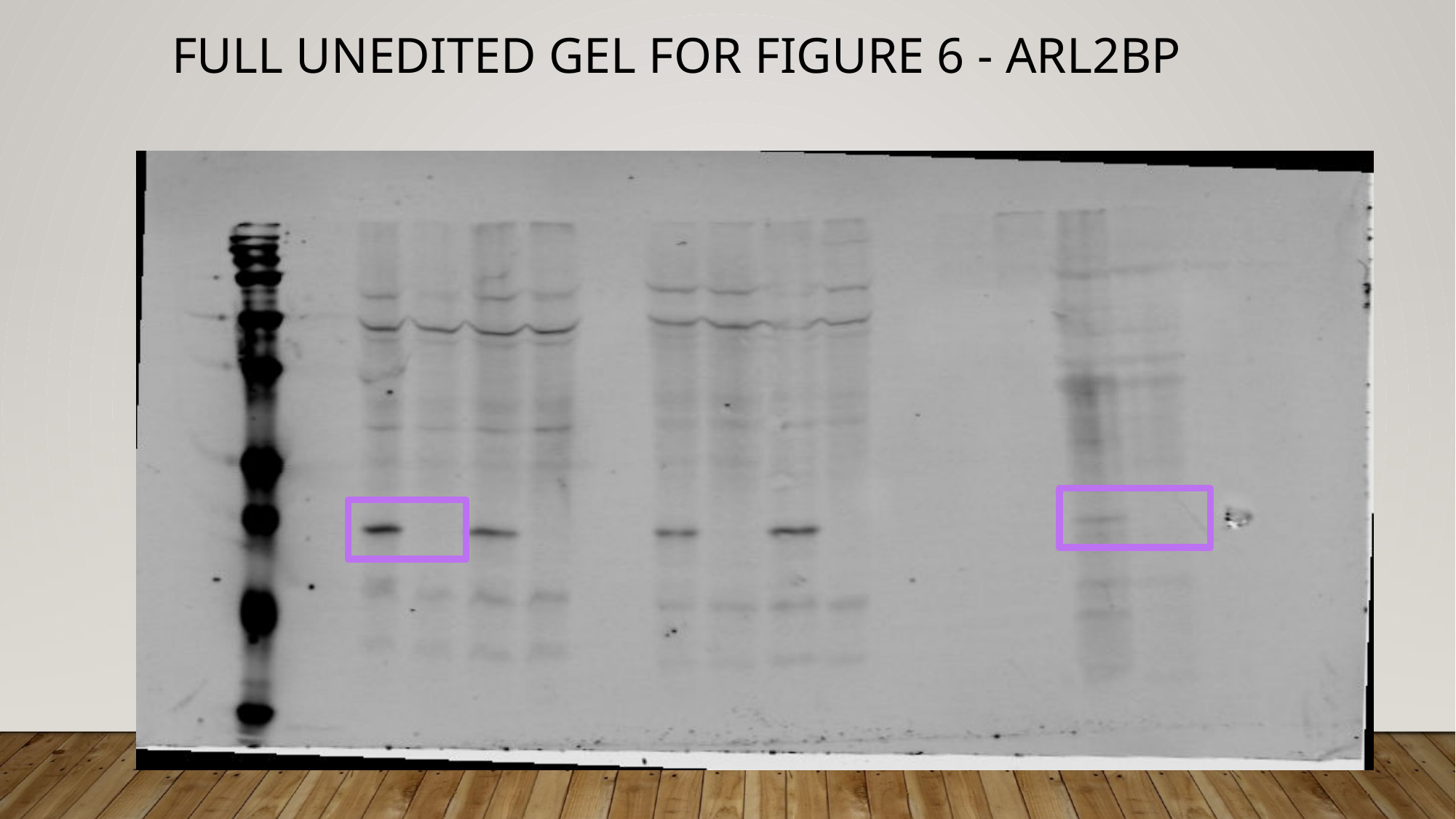

# Full unedited gel for Figure 6 - ARL2BP

## Slide 7
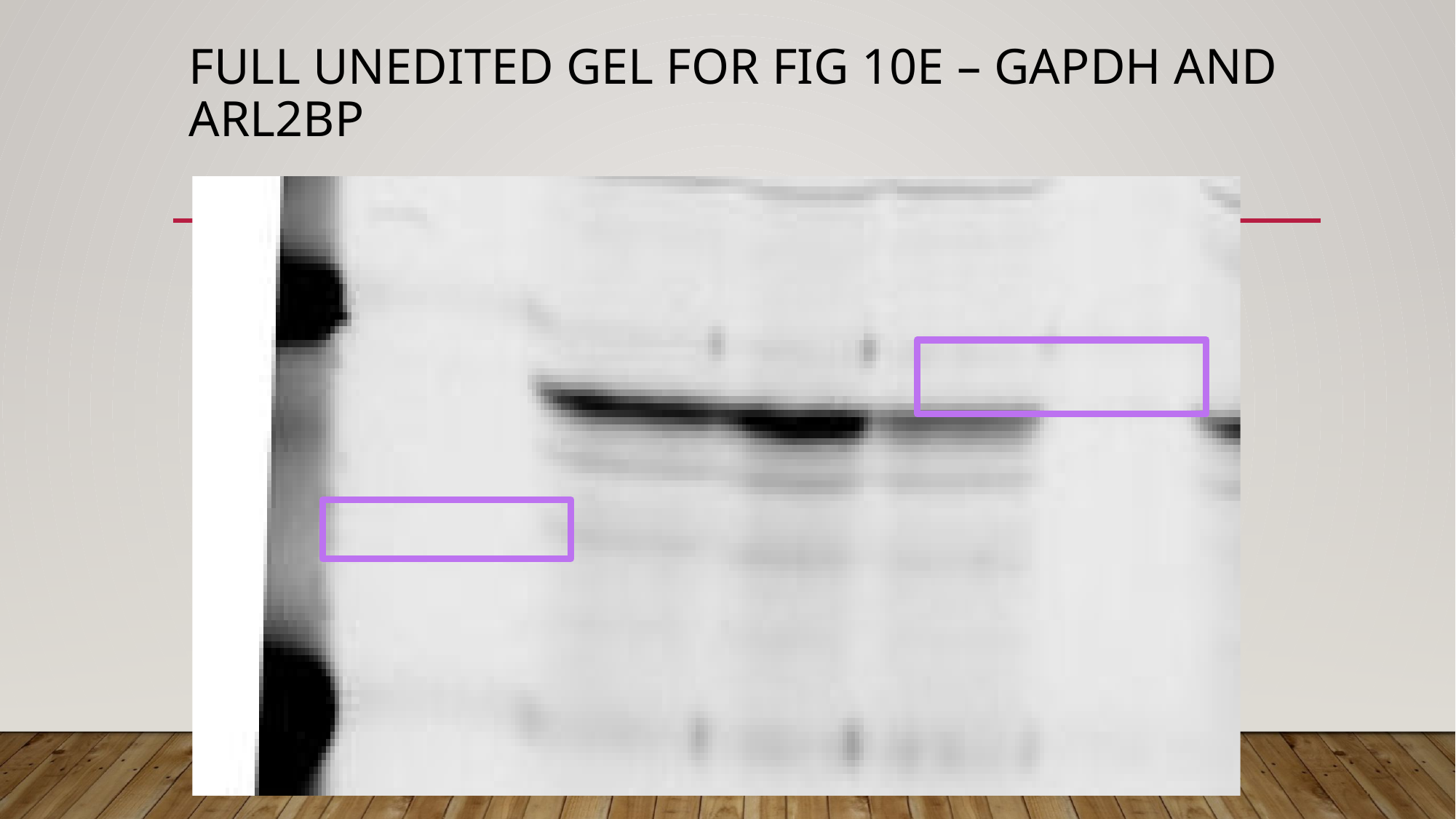

# Full unedited gel for Fig 10E – GAPDH and ARL2BP
